# Dynamics of antiphase bursting modulated by the inhibitory synaptic and hyperpolarization-activated cation currents

**DOI:** 10.3389/fncom.2024.1303925

**Published:** 2024-02-09

**Authors:** Linan Guan, Huaguang Gu, Xinjing Zhang

**Affiliations:** ^1^School of Mathematics and Statistics, North China University of Water Resources and Electric Power, Zhengzhou, China; ^2^School of Aerospace Engineering and Applied Mechanics, Tongji University, Shanghai, China

**Keywords:** antiphase bursting, bifurcation, *I*_h_ current, inhibitory synaptic current, fast-slow dissection method

## Abstract

Antiphase bursting related to the rhythmic motor behavior exhibits complex dynamics modulated by the inhibitory synaptic current (*I*_syn_), especially in the presence of the hyperpolarization-activated cation current (*I*_h_). In the present paper, the dynamics of antiphase bursting modulated by the *I*_h_ and *I*_syn_ is studied in three aspects with a theoretical model. Firstly, the *I*_syn_ and the slow *I*_h_ with strong strength are the identified to be the necessary conditions for the antiphase bursting. The dependence of the antiphase bursting on the two currents is different for low (escape mode) and high (release mode) threshold voltages (*V*_th_) of the inhibitory synapse. Secondly, more detailed co-regulations of the two currents to induce opposite changes of the bursting period are obtained. For the escape mode, increase of the *I*_h_ induces elevated membrane potential of the silence inhibited by a strong *I*_syn_ and shortened silence duration to go beyond *V*_th_, resulting in reduced bursting period. For the release mode, increase of the *I*_h_ induces elevated tough value of the former part of the burst modulated by a nearly zero *I*_syn_ and lengthen burst duration to fall below *V*_th_, resulting in prolonged bursting period. Finally, the fast-slow dynamics of the antiphase bursting are acquired. Using one-and two-parameter bifurcations of the fast subsystem of a single neuron, the burst of the antiphase bursting is related to the stable limit cycle, and the silence modulated by a strong *I*_syn_ to the stable equilibrium to a certain extent. The *I*_h_ mainly modulates the dynamics within the burst and quiescent state. Furthermore, with the fast subsystem of the coupled neurons, the silence is associated with the unstable equilibrium point. The results present theoretical explanations to the changes in the bursting period and fast-slow dynamics of the antiphase bursting modulated by the *I*_syn_ and *I*_h_, which is helpful for understanding the antiphase bursting and modulating rhythmic motor patterns.

## 1 Introduction

Rhythmic motor behavior, such as walking, swimming, flying, breathing, and chewing, are important for vertebrates and invertebrates (Marder and Calabrese, 1996; Marder and Bucher, 2001; Katz, 2016; Kiehn, 2016; Sakurai and Katz, 2016; Lu et al., 2022). Motor behaviors are modulated by the rhythmic patterns generated in the central pattern generators (CPGs) or neuronal circuits with inhibitory coupling, such as the stomatogastric ganglion (STG) in *Cancer borealis* or *Panulirus interruptus*. The triphasic rhythm generated by three neurons of the pyloric network of the STG controls the digestive function (Selverston, 2005; Szücs et al., 2009; Zhu et al., 2016). The bursting period, burst duration, duty cycle, spike frequency, spikes per burst of rhythmic bursting activity are essential factors to modulate rhythmic behaviors (Daun et al., 2009; Coleman et al., 2013; Kueh et al., 2016). For instance, shortened period of bursting in the leech heart interneurons speeds up the heartbeat (Tobin and Calabrese, 2005). Especially, rhythmic patterns have been widely used to control the motion of robot (Ijspeert, 2008; Habu et al., 2019; Fukuoka et al., 2022). Then, identification of modulations such as inhibitory currents of synapses and ionic currents of neurons to the rhythmic motion is an important issue (Marder and Bucher, 2007; Grashow et al., 2009; O’Leary et al., 2014; Kueh et al., 2016).

Inhibitory synaptic current (*I*_syn_) modulates the dynamical behaviors of two neurons with reciprocal inhibition coupling (Alaçam and Shilnikov, 2015; Sakurai and Katz, 2016; Baruzzi et al., 2020; Onasch and Gjorgjieva, 2020), such as the antiphase rhythm composed of firing (active) phase and silence (inhibited) phase. For example, changes in durations of the two phases underlie various rhythmic behaviors, such as breathing fast or slow (Peña et al., 2004; Baertsch et al., 2018). The antiphase rhythmic patterns can be roughly classified into two typical mechanisms: escape and release (Wang and Rinzel, 1992; Skinner et al., 1994). In this paper, they are called escape mode and release mode, which correspond to the low and high voltage thresholds (*V*_th_) of the inhibitory synapse, respectively. For the escape mode, the low membrane potential during the silence phase of neuron 1 can depolarize to go beyond *V*_th_ and become firing, which terminates the firing phase of neuron 2 (Skinner et al., 1994). For the release mode, the membrane potential of the firing phase of neuron 1 falls below *V*_th_ to reduce the inhibitory synaptic current (*I*_syn_) outputted to the silence phase of neuron 2, then, the silence phase of neuron 2 transits to the firing phase. The firing phase in Wang and Rinzel (1992) is not burst but a spike due to the relatively simple ionic currents of the neuron model. For the bursting neuron model such as the Hindmarsh–Rose model, inhibitory coupling can induce antiphase bursting (Yao et al., 2013). Other currents such as the calcium current and pump current play important roles to modulate the rhythmic patterns containing the antiphase bursting (Olsen et al., 1995; Olypher et al., 2006; Simoni and DeWeerth, 2006; Doloc-Mihu and Calabrese, 2011; Doloc-Mihu Anca and Calabrese, 2014; Li et al., 2018). More interestingly, for the spiking neuron with hyperpolarization-activated cation current (*I*_h_), inhibitory coupling can induce antiphase bursting (Sharp et al., 1996).

The *I*_h_, which has been identified in many different types of neurons (Robinson and Siegelbaum, 2003; He et al., 2014; Xu et al., 2017; Dashevskiy and Cymbalyuk, 2018), is known to regulate rhythmic behavior and excitability (Lüthi and McCormick, 1998; Sorensen et al., 2004; Biel et al., 2009; Wahl-Schott and Biel, 2009; Datunashvili et al., 2018). Especially, as a neuron is subjected to negative, hyperpolarization, or inhibitory stimulation, the membrane potential becomes lower, which can activate the positive *I*_h_ to promote the neuronal excitability and evoke action potentials. Then, the *I*_h_ and the co-regulations of the inhibitory synaptic current *I*_syn_ and the *I*_h_ play important roles in modulating the antiphase bursting in many different types of neurons (Sorensen et al., 2004; Grashow et al., 2009; Ellingson et al., 2021; Morozova et al., 2022). For example, in pairs of the leech heart interneurons with mutually inhibitory coupling, increase of the *I*_h_ current induces the bursting period of the antiphase bursting decreased to speed up the heartbeat (Tobin and Calabrese, 2005). Especially, in the mutually inhibitory circuit containing two gastric mill (GM) neurons of the STG of the crab, many rhythm patterns that does not contain the antiphase bursting appear in the absence of the *I*_h_, whereas introduction of the *I*_h_ induces stable antiphase bursting (Sharp et al., 1996; Morozova et al., 2022). Unfortunately, the parameter regions of the *I*_h_ and *I*_syn_ for the antiphase bursting and other rhythm patterns, and the changes of the parameter region with respect to changes of *V*_th_ (the synaptic threshold voltage), are still unclear, which are studied in the present paper.

Then, complex dynamics of two mechanisms of the antiphase bursting modulated by the *I*_h_ are studied (Sharp et al., 1996; Clewley, 2011; Morozova et al., 2022). With increasing the conductance (*g*_h_) of the *I*_h_, the bursting period decreases and increases, respectively, for the escape mode and release mode, exhibiting opposite changes (Sharp et al., 1996; Morozova et al., 2022). More complex, a mixture mechanism locating between the escape mode and release mode is observed in a recent study (Morozova et al., 2022), and the robustness of the two manners is studied. Especially, the opposite changes of the bursting period with respect to the *I*_h_ also provide a convenient and feasible measure to generate rhythmic bursting patterns with various periods, which may be used as a potential candidate to control motion of the robot. Although experimental investigations, there are lack of theoretical studies. In addition, although the alternation between the burst and the silence of the antiphase bursting for the escape mode and release mode is presented (Sharp et al., 1996; Morozova et al., 2022), the co-regulations of the *I*_h_ and *I*_syn_ in modulating the opposite changes of the bursting period for the two manners have not been explained very well. Then, reproduction of the antiphase bursting for the escape, release, and mixture modes in the simulations, and identification of the roles of the *I*_h_ and *I*_syn_ in the opposite changes of bursting period for the escape mode and release mode, are important questions, which are answered in the present paper.

In addition, according to the fast-slow dynamics of the bursting (Izhikevich, 2000; Li Y. et al., 2021; Ma et al., 2021), a neuron model with bursting behavior contains a fast subsystem and slow variables (Wang et al., 2021; Yuan et al., 2021), and the fast-slow analysis is effective to analyze the dynamics of the bursting and spiking modulated by one slow variable (Lü et al., 2019). After obtaining the bifurcations of the fast subsystem with the slow variable taken as a bifurcation parameter, the burst phase and quiescent (silence) phase of the bursting pattern are, respectively, related to the stable limit cycle behavior (spiking) and stable equilibrium point of the fast subsystem (Belykh and Shilnikov, 2008). However, a spiking is only related to the stale limit cycle instead of the stable equilibrium point. Recently, fast-slow analysis with two slow variables is proposed. Up to now, fast-slow analysis has seldom been used to analyze the bursting from the coupled neurons. In the experimental study (Sharp et al., 1996), for the isolated neurons with a slow *I*_h_ generating the spiking instead of the antiphase bursting, the antiphase bursting appears after the inhibitory coupling. Unfortunately, the mechanism for the transition from the spiking to the antiphase bursting and the fast-slow dynamics of the antiphase bursting, which are modulated by the *I*_syn_ and *I*_h_, are still unclear. Then, the fast-slow dynamics of the antiphase bursting, the roles of the *I*_syn_ and *I*_h_ in the alternation between the silence and burst phases, and the roles of the two currents within the burst and silence phases are studied in the present paper.

In the present paper, the questions mentioned above for the dynamics of the antiphase bursting are answered in a theoretical model of two neurons with mutually inhibitory coupling. Firstly, the roles of the slow *I*_h_ to ensure the formation of the antiphase bursting, and the different influences of the *I*_h_ and *I*_syn_ on the antiphase bursting at different values of *V*_th_, are obtained. The *I*_h_ is identified to be the necessary condition for the antiphase bursting, closely matching the experimental observation (Sharp et al., 1996). At different levels of *V*_th_, the dependence of the antiphase bursting and other rhythm patterns on the *I*_h_ and *I*_syn_ is different, which presents the parameter region of the antiphase bursting. Secondly, the opposite changes of the antiphase bursting for the escape mode and release mode, respectively, corresponding to low and high *V*_th_, and a mixture mode for a middle *V*_th_, are reproduced in the simulations, closely matching the experimental observations (Morozova et al., 2022). Furthermore, the roles of the *I*_h_ and *I*_syn_ in modulating the opposite changes of bursting period for the escape mode and release mode are obtained. For the escape mode, increase of the *I*_h_ induces elevated membrane potential of the silence inhibited by a strong *I*_syn_, which reduces the silence duration to go beyond *V*_th_, resulting in reduced bursting period. For the release mode, with increasing *I*_h_, the tough value of the burst elevates, then, the burst duration with tough value higher than *V*_th_ is lengthened, meanwhile, enhanced *I*_syn_ is outputted to the partner neuron to prolong the silence period, resulting in prolonged bursting period. Finally, the fast-slow dynamics of antiphase bursting modulated by the *I*_h_ and *I*_syn_ are acquired. Using the one-parameter and two-parameter bifurcations of the fast subsystem of a single neuron, the burst of the antiphase bursting is related to the stable limit cycle modulated by a weak *I*_syn_, and the silence modulated by a strong *I*_syn_ to the stable equilibrium point to a certain extent. The *I*_h_ mainly modulates the dynamics within the burst and silence. Furthermore, using the fast subsystem of the coupled neurons, the silence is related to the unstable equilibrium point. The results present theoretical explanations to the variations in the bursting period and fast-slow dynamics of the antiphase bursting modulated by the *I*_syn_ and *I*_h_, which is helpful for understanding the antiphase bursting. Especially, a potential and feasible measure to modulate the rhythmic motion via modulations to only two factors (the *I*_h_ and *I*_syn_) to obtain different bursting patterns with various periods.

## 2 Models and methods

### 2.1 Single neuron model

Stomatogastric ganglion (STG) and leech heart interneurons with mutually inhibitory coupling are often used to study antiphase bursting modulated by the *I*_syn_ and *I*_h_. For example, the experiments on the escape mode and release mode are performed on two gastric mill (GM) neurons of the STG (Morozova et al., 2022). Although the STG is different from the leech heart interneurons, antiphase bursting of the STG and leech heart interneurons modulated by both the *I*_syn_ and *I*_h_ should have same dynamics in some aspects. Then, in the present paper, the model of the leech heart interneuron is used as representative, and the commonly used parameter values are adopted (Hill et al., 2001; Belykh and Shilnikov, 2008).

The single leech neuronal model (Barnett and Cymbalyuk, 2014) contains five currents: a sodium ion current (*I*_Na_), a potassium ion current (*I*_K_), a leakage current (*I*_L_), a polarization current (*I*_pol_), and an *I*_h_ current, described as follows:

(1) $$C\frac{dV}{dt}=-\left[\begin{array}{l} g_{Na}f_{\infty }{\left(-150,0.0305,V\right)}^{3}h_{Na}\left(V-E_{Na}\right)+\\ g_{K}m_{K}^{2}\left(V-E_{K}\right)+g_{h}m_{h}^{2}\left(V-E_{h}\right)+g_{L}\left(V-E_{L}\right) \end{array}\right]+I_{pol}$$

(2) $$\frac{dh_{Na}}{dt}=\frac{f_{\infty }\left(500,0.0325,V\right)-h_{Na}}{\tau_{Na}}$$

(3) $$\frac{dm_{K}}{dt}=\frac{f_{\infty }\left(-83,0.008,V\right)-m_{K}}{\tau_{K}}$$

(4) $$\frac{dm_{h}}{dt}=\frac{1/\left[1+2e^{180\left(V+\theta_{h}\right)}+e^{500\left(V+\theta_{h}\right)}\right]-m_{h}}{\tau_{h}}$$

where *V* represents the membrane potential, *h*_Na_, *m*_K_, and *m*_h_ denote the gating variables to describe the inactivation of the sodium (Na^+^) current, the activation of the potassium (K^+^) current, and the inactivation of the *I*_h_ current, respectively. The parameter *C* is the membrane capacitance, and the parameters *g*_Na_, *g*_K_, *g*_L_, and *g*_h_ are the corresponding maximal conductances, *E*_Na_, *E*_K_, *E*_L_, and *E*_h_ are the reversal potentials, and *τ*_Na_, *τ*_K_, and *τ*_h_ are the relaxation time. The parameter *θ*_h_ represents the activation potential of the variable *m*_h_. The function $f_{\infty }\left(x,y,z\right)=1/\left(1+e^{x\left(y+z\right)}\right)$ .

The parameter values are *C* = 0.5 nF, *g*_Na_ = 200 nS, *g*_K_ = 30 nS, *g*_L_ = 8 nS, *E*_Na_ = 0.045 V, *E*_K_ = −0.07 V, *E*_L_ = −0.046 V, *E*_h_ = −0.021 V, *τ*_Na_ = 0.0405 s, *τ*_K_ = 0.9 s, *τ*_h_ = 0.1 s, *θ*_h_ = 0.04 V. *I*_pol_ and *g*_h_ are the control parameters.

### 2.2 Neurons with reciprocally inhibitory coupling

Two leech neurons 1 and 2 are coupled via the inhibitory synapses. Except for other equations, the two equations of membrane potentials containing the inhibitory coupling currents are described as follows:

(5) $$C\frac{dV_{i}}{dt}=-\left[\begin{array}{l} g_{Na}f_{\infty }{\left(-150,0.0305,V_{i}\right)}^{3}h_{Na,i}\\ \left(V_{i}-E_{Na}\right)+g_{K}m_{K,i}^{2}\left(V_{i}-E_{K}\right)\\ +g_{h}m_{h,i}^{2}\left(V_{i}-E_{h}\right)+g_{L}\left(V_{i}-E_{L}\right) \end{array}\right]+I_{pol}+I_{syn,i}$$

(6) $$\frac{dh_{Na,i}}{dt}=\frac{f_{\infty }\left(500,0.0325,V_{i}\right)-h_{Na,i}}{\tau_{Na}}$$

(7) $$\frac{dm_{K,i}}{dt}=\frac{f_{\infty }\left(-83,0.008,V_{i}\right)-m_{K,i}}{\tau_{K}}$$

(8) $$\frac{dm_{h,i}}{dt}=\frac{1/\left[1+2e^{180\left(V_{i}+\theta_{h}\right)}+e^{500\left(V_{i}+\theta_{h}\right)}\right]-m_{h,i}}{\tau_{h}}$$

(9) $$\frac{dS_{i}}{dt}=\alpha \left(1-S_{i}\right)S_{\infty }\left(V_{j}\right)-\beta S_{i}$$

where *V_i_* is the membrane potentials of the neurons *i* (*i* = 1, 2). *I*_syn,*i*_ is the synaptic current received by the neuron *i* (*i* = 1, 2). The *I*_syn,*i*_ is described as follows:

$$I_{syn,i}=g_{syn}S_{i}\left(V_{syn}-V_{i}\right)\text{,}$$

where *g*_syn_ represents the maximal synaptic conductance of the inhibitory synapse, *S* is the variable to describe the instantaneous synaptic activation, *V*_syn_ denotes the synaptic reversal potential. $S_{\infty }\left(V_{j}\right)=\frac{1}{1+e^{-1000\left(V_{j}-V_{th}\right)}}$ is the steady state of the synaptic activation function, and *V_j_* is the presynaptic voltage for the neuron *i*, with *j* = 1 for *i* = 2 and *j* = 2 for *i* = 1. The parameters α and β denote the opening and closing rates of the synaptic current channels, respectively. *V*_th_ containing in $S_{\infty }\left(V_{j}\right)=\frac{1}{1+e^{-1000\left(V_{j}-V_{th}\right)}}$ is the synaptic threshold voltage. In the present paper, *V*_syn_ = −0.0625 V, *α* = 1,000, and *β* = 100. *g*_syn_ and *V*_th_ are the control parameters.

As can be found from Eqs. 5–9, the parameters for the neuron 1 and neuron 2 are the same, resulting in that the neuron 1 and neuron 2 are symmetrical. The system remains unchanged if “1” and “2” in Eqs. 5, 6 exchange each other. Thus, the neuron 1 and neuron 2 exhibit symmetrical behaviors.

For the real neurons and synapses studied in the experiments (Sharp et al., 1996; Morozova et al., 2022), their parameter values should be heterogeneous. However, there are many parameters for the two neurons and synapses, then, too many calculations should be performed if heterogeneous parameter values are considered. In the present paper, the neurons 1 and 2 are assigned to be the same parameter values, i.e., the neuron 1 and 2 are symmetrical, which is the first step for the studies to the antiphase bursting modulated by the *I*_h_ and *I*_syn_. In future, heterogeneous parameter values for the two neurons will be considered. In addition, fast or slow dynamics of the *I*_h_ and *I*_syn_ are important factors to modulate the dynamics of the antiphase bursting. In the present paper, slow *I*_h_ is considered to ensure the appearance of the antiphase bursting, and fast decay of *I*_syn_ is considered to ensure that the bursting is modulated by only one slow variable and the bursting can be effectively analyzed by the fast-slow dissection method. In future, slow decay of *I*_syn_ will be studied and there may be very complex dynamics for the antiphase bursting.

### 2.3 Methods

The equations of the theoretical models are integrated with Euler method with time step 0.0001 s. The bifurcations are acquired with the software XPPAUT. See “XPPAUT code” in the Supplementary material for the relevant program code *I*_pol_ and *m*_h_ are chosen as the bifurcation parameters.

## 3 Results

### 3.1 Antiphase bursting of the coupled neurons with the *I*_h_

#### 3.1.1 Four rhythm patterns in the absence of the *I*_h_ current

**Figure 1:**
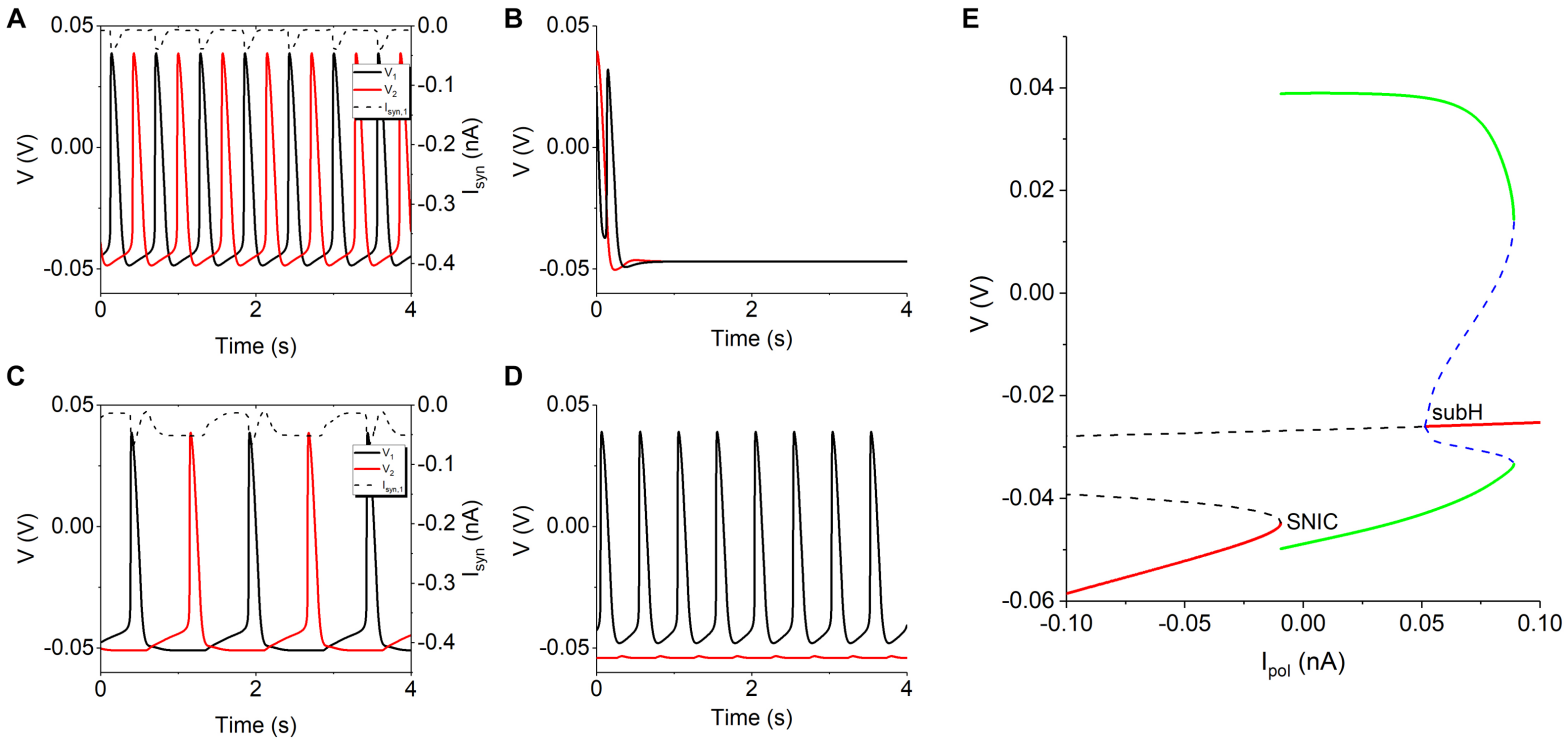
The four rhythm patterns of model without *I*_h_ current at different *g*_syn_ values. **(A)** Double-spiking pattern for *g*_syn_ = 0.5 nS. **(B)** Double-silence pattern for *g*_syn_ = 2 nS. **(C)** Antiphase spiking for *g*_syn_ = 5 nS. **(D)** Pattern of spiking and subthreshold oscillation for *g*_syn_ = 10 nS. Synaptic threshold *V*_th_ = −0.047 V. **(E)** Bifurcations of an isolated neuron to explain the rhythm patterns in panels **(A,C)**.

In the absence of the *I*_h_, the two neurons with the inhibitory coupling produce four patterns of electrical activity, as shown in Figure 1. Unfortunately, antiphase bursting does not appear. The solid black and solid red curves represent the voltage of the neuron 1 and neuron 2, respectively, and the dashed curve denotes the inhibitory synaptic current *I*_syn_. For a small coupling strength such as *g*_syn_ = 0.5 nS, either neuron exhibits spiking similar to that of the isolated neuron, as shown in Figure 1A, which is called double-spiking pattern in this article. For *g*_syn_ = 2 nS, the two neurons inhibit each other at first due to the increase of the synaptic current, then the behavior of the two neurons changes to silence state, as depicted in Figure 1B, called double-silence pattern. With further increasing *g*_syn_ to 5 nS, antiphase spiking appears, induced by the mutual inhibitions between the two neurons, as shown in Figure 1C. As *g*_syn_ becomes strong such as 10 nS, the inhibitory current from the neuron 1 to the neuron 2 is strong enough, resulting in that spiking appears for neuron 1 and subthreshold oscillation for neuron 2, as illustrated in Figure 1D, which is called pattern of spiking and subthreshold oscillation. Such a symmetrical behavior is caused by different initial values of the two neurons.

The rhythm patterns in panels (A,C) are different, which can be explained with the inhibitory coupling current and the bifurcations of the isolated neuron. For the panel (A), the inhibitory synaptic current (dashed curve for neuron 1) between two continuous spikes is small, weaker than −0.009485 nA, as shown in Figure 1A, which can play a weak role and then can seldom influence the spiking behavior. For the panel (C), the inhibitory synaptic current (dashed curve for neuron 1) between two continuous spikes is strong, stronger than −0.009485 nA for a relatively long time, which can inhibit a spike to from a relatively long interspike interval (ISI). As shown in Figure 1E, there is a saddle-node bifurcation on an invariant cycle (SNIC) at *I*_pol_ = −0.009485 nA for the isolated neuron. Via the SNIC bifurcation, the resting state (left red, stable node) changes to the spiking (green curves). The resting state appears for *I*_pol_ < −0.009485 nA. Then, *I*_syn_ weaker than −0.009485 nA in panel (A) cannot go beyond the bifurcation, forming the silence, whereas *I*_syn_ stronger than −0.009485 nA for a relatively long time in the panel (C) can induce silence corresponding to the resting state and appearing between two continuous spikes, resulting in a long ISI. Other bifurcations are not related to the results of the present paper (not addressed here).

#### 3.1.2 Antiphase bursting pattern in the presence of the slow *I*_h_

**Figure 2:**
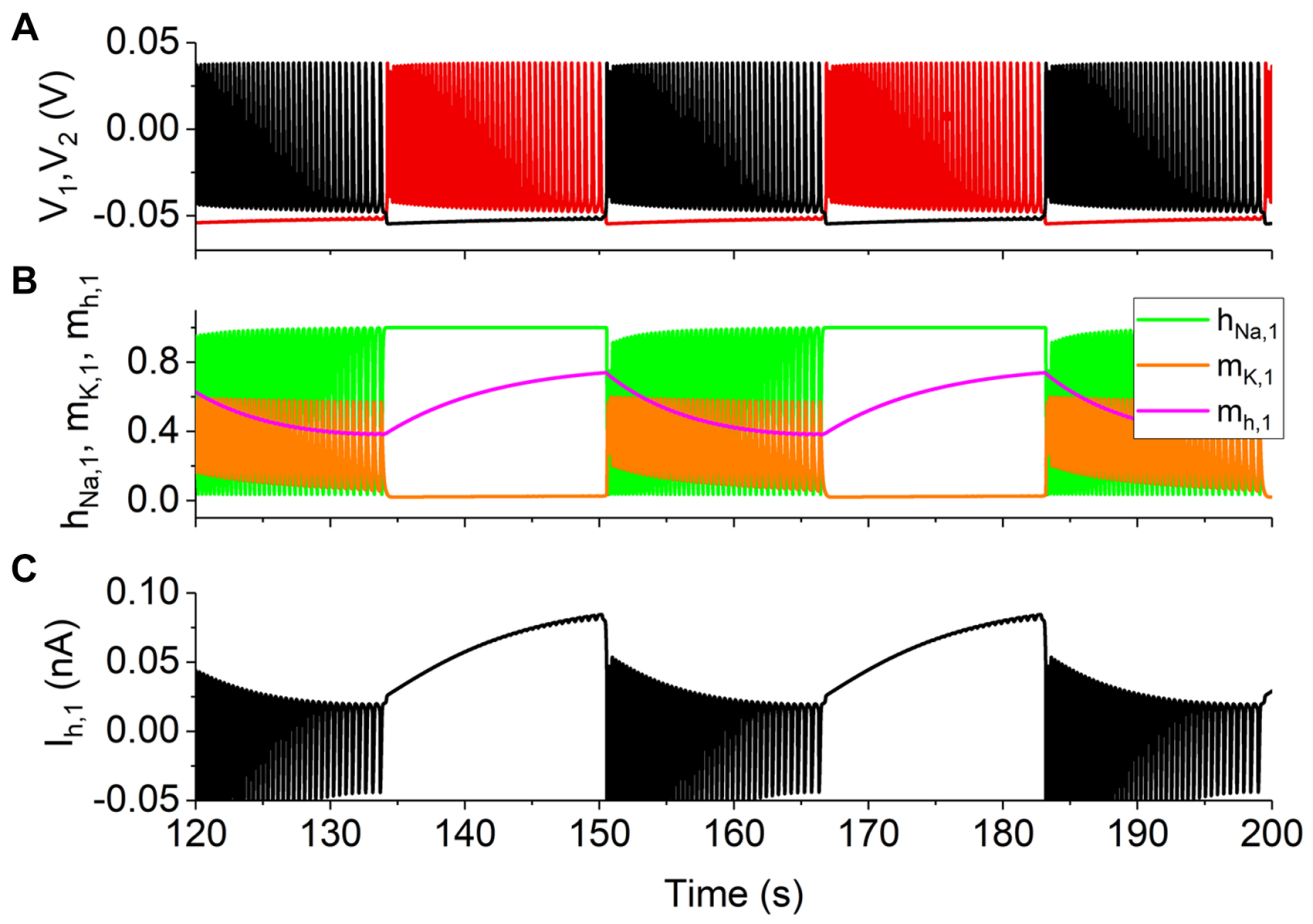
The slow *I*_h_ for the antiphase bursting with *g*_syn_ = 15 nS, *g*_h_ = 5 nS, and *V*_th_ = −0.047 V. **(A)** Spike trains with black curve for neuron 1 and red curve for neuron 2. **(B)** Variables *h*_Na_ (green), *m*_K_ (orange), and *m*_h_ (magenta) with slow dynamics for the neuron 1 (black). **(C)** *I*_h_ for the neuron 1 (black).

In the presence of the slow *I*_h_, in addition to the four electrical activity patterns mentioned above, the antiphase bursting activity appears, as shown in Figure 2A, black representing the neuron 1 and red denoting the neuron 2, which is consistent with the experimental results in Sharp et al. (1996). It is well-known that the bursting behavior is modulated by slow variables (Berry et al., 2022; Xing et al., 2022). The gating variable *m*_h_ of the *I*_h_ is a slow variable, as shown by the magenta curve in Figure 2B. The variables *h*_Na_ (green) and *m*_K_ (orange) of the neuron 1 are shown in Figure 2B, and the *I*_h_ of the neuron 1 is shown in Figure 2C. Obviously, during the burst, variables *h*_Na_ (green) and *m*_K_ (orange) oscillate fast, similarly to *V*, whereas *m*_h_ (magenta) changes slowly and gradually, showing that *m*_h_ is the slow variable to ensure the bursting activity.

#### 3.1.3 The dependence of the antiphase bursting on *g*_h_ and *g*_syn_ for different values of *V*_th_

**Figure 3:**
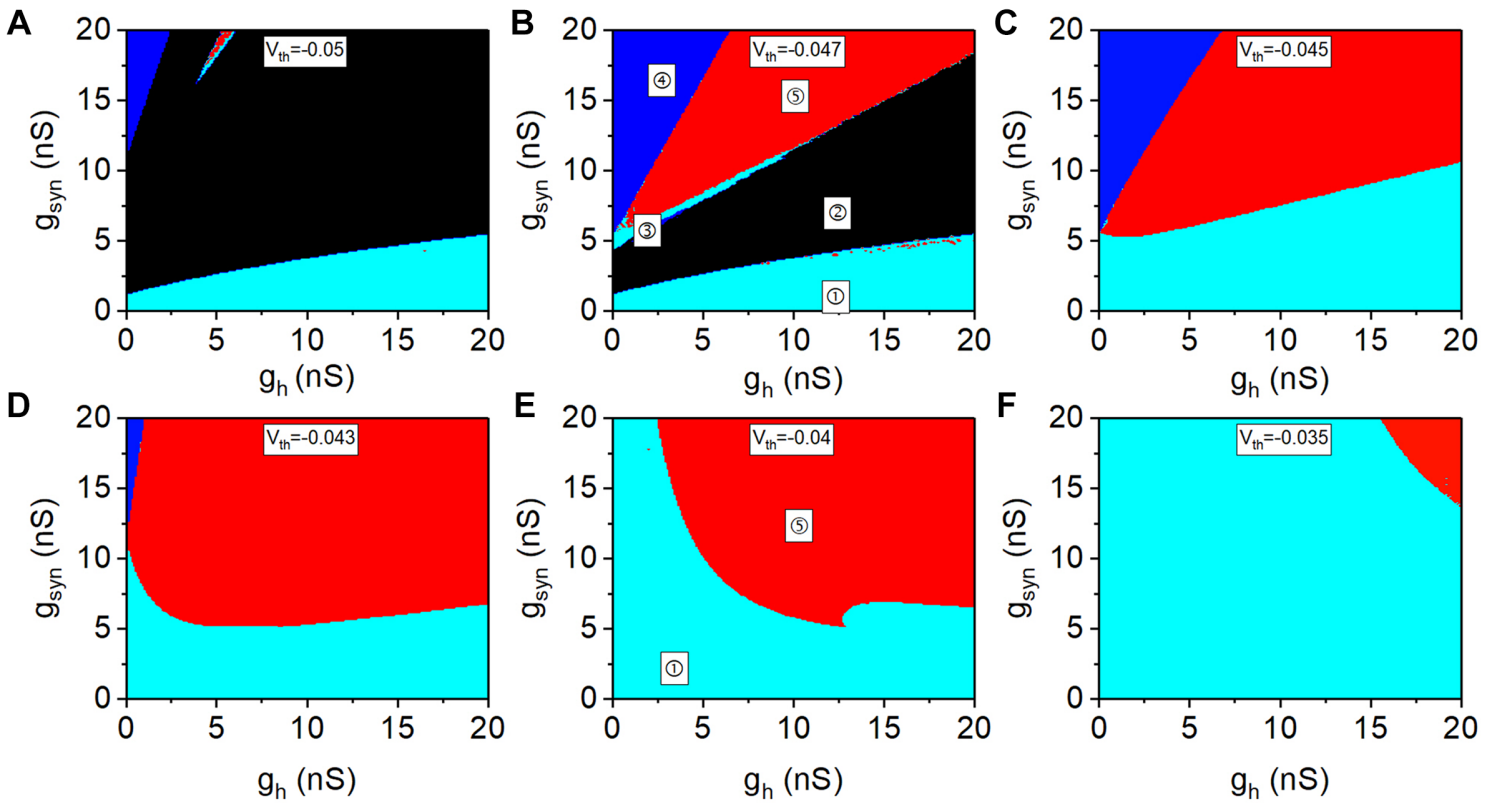
Distribution of different rhythm patterns in plane (*g*_h_, *g*_syn_) at different *V*_th_ values. **(A)** *V*_th_ = −0.05 V; **(B)** *V*_th_ = −0.047 V; **(C)** *V*_th_ = −0.045 V; **(D)** *V*_th_ = −0.043 V; **(E)** *V*_th_ = −0.04 V; **(F)** *V*_th_ = −0.035 V. The areas marked with ①, ②, ③, ④, and ⑤ represent the double-spiking pattern, double-silence pattern, antiphase spiking pattern, antiphase bursting pattern, and pattern of spiking and subthreshold oscillation.

The distribution of the five rhythm patterns at different values of *V*_th_ in the plane (*g*_h_, *g*_syn_) are shown in Figures 3A–F. The lower cyan area (①), black area (②), upper cyan area (③), red area (④), and blue area (⑤) represent the double-spiking pattern, double-silence pattern, antiphase spiking pattern, antiphase bursting pattern, and pattern of spiking and subthreshold oscillation, respectively. In Ellingson et al. (2021), *V*_th_ determines the escape mode and release mode for the antiphase bursting. The value of *V*_th_ determines the level of inhibitory synaptic current (please refer to Supplementary Figure S1). With increasing *V*_th_, the inhibitory synaptic current becomes weak. Therefore, the distribution of the rhythm patterns on plane (*g*_h_, *g*_syn_) at different values of *V*_th_ are different, as shown in Figure 3. The results present more detailed relationships between the different rhythm patterns (Sharp et al., 1996) show as follows:

1. When *g*_h_ is zero or small, no antiphase bursting (red) appears. Antiphase bursting (red area) occurs for positive *g*_h_ values, which is consistent with the experimental results in Wahl-Schott and Biel (2009).
2. The red region for the antiphase bursting exhibits large *g*_syn_ and *g*_h_, showing that the antiphase bursting appears for strong *I*_h_ and strong *I*_syn_.
3. The red region for the antiphase bursting exhibits different characteristics for lower *V*_th_ [panels (A–D) for −0.05 V, −0.047 V, −0.045 V, and *V*_th_ = −0.043 V, respectively] and higher *V*_th_ [panels (E,F) for −0.04 V and −0.035 V, respectively]. As *V*_th_ increases, the red parameter region for the antiphase bursting becomes large for a lower *V*_th_, and becomes small for a higher *V*_th_. The borders of red region (the antiphase bursting) for low *V*_th_ exhibit shapes different from those of high *V*_th_. Then, different dynamics for a lower *V*_th_ (−0.047 V as representative) and a higher *V*_th_ (−0.04 V as representative) are studied in the following paragraphs.

### 3.2 The roles of the *I*_syn_ and *I*_h_ for the escape mode and release mode of the antiphase bursting

The antiphase bursting for escape mode (*V*_th_ = −0.047 V as representative) and release mode (*V*_th_ = −0.04 V as representative) is studied in the present subsection. As mentioned above, the neuron 1 and neuron 2 exhibit symmetrical behaviors. Then, the inhibited (silence) phase of the neuron 1 (black) corresponds to the active (burst) phase of the neuron 2 (red), and the burst duration of the neuron 1 equals the duration of the silence phase of the neuron 2, and vice verse, as shown in Supplementary Figure S2.

#### 3.2.1 Distributions of the bursting period in plane (*g*_h_, *g*_syn_) for the three manners

**Figure 4:**
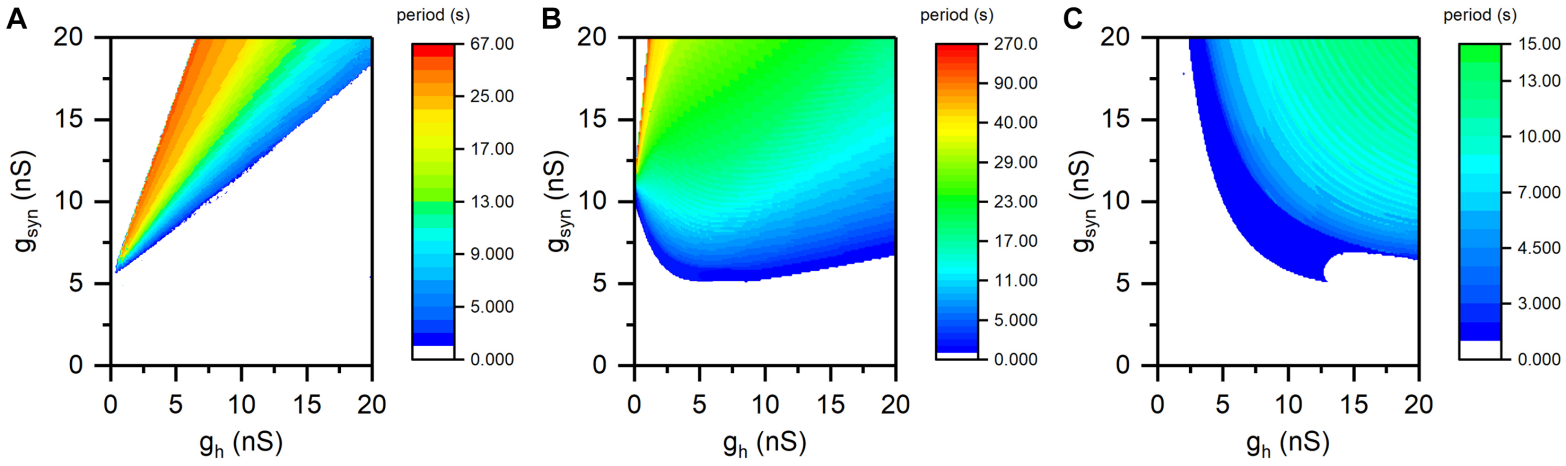
The distribution of period of antiphase bursting in plane (*g*_h_, g_syn_). **(A)** *V*_th_ = −0.047 V for the escape mode; **(B)** *V*_th_ = −0.043 V for the mixture mode; **(C)** *V*_th_ = −0.040 V for the release mode. Color scale represents the value of bursting period.

The period of the antiphase bursting shows opposite changes at different synaptic thresholds, as shown in Supplementary Figure S2. The distributions of the bursting period on two-parameter plane (*g*_h_, *g*_syn_) at different *V*_th_ values are shown in Figure 4. The antiphase bursting appears in the colorful region and the color scale represents the value of the bursting period, and the blank area represents other rhythm patterns. For different *V*_th_ values, the bursting period exhibits different changes with respect to *g*_h_.

For *V*_th_ = −0.047 V (escape mode), as illustrated in Figure 4A, the bursting period decreases with the increase of *g*_h_ for all values of *g*_syn_, which presents the simulation results to the experimental observations in Sharp et al. (1996) and Morozova et al. (2022). And the maximum bursting period exceeds 60 s.

When *V*_th_ = −0.043 V, the change of bursting period with increasing *g*_h_ is complex, as shown in Figure 4B. As *g*_syn_ is approximately less than 11 nS, the bursting period increases at first and then decreases. When *g*_syn_ is approximately greater than 11 nS, the bursting period decreases. Such a manner does not correspond to the typical escape mode or release mode, but to the mixture mode observed in the experiment (Morozova et al., 2022).

For *V*_th_ = −0.04 V (release mode), the period of bursting increases with increasing *g*_h_ for all *g*_syn_ values, as depicted in Figure 4C, presenting simulation results to the experimental observations reported in Sharp et al. (1996) and Morozova et al. (2022). In addition, the maximum burst period at *V*_th_ = −0.04 V is less than 15 s, which is significantly lower than the bursting period for *V*_th_ = −0.047 V.

In addition, the bursting period increases with increasing *g*_syn_ for different *V*_th_ and *g*_h_ values, which is easy to be understand. The larger the *g*_syn_ is, the stronger the *I*_syn_ is, and the longer the silence duration is. Then, burst duration and bursting period increases with increasing *g*_syn_, which is not studied in the present paper.

#### 3.2.2 Co-regulations of the two currents for the escape mode

**Figure 5:**
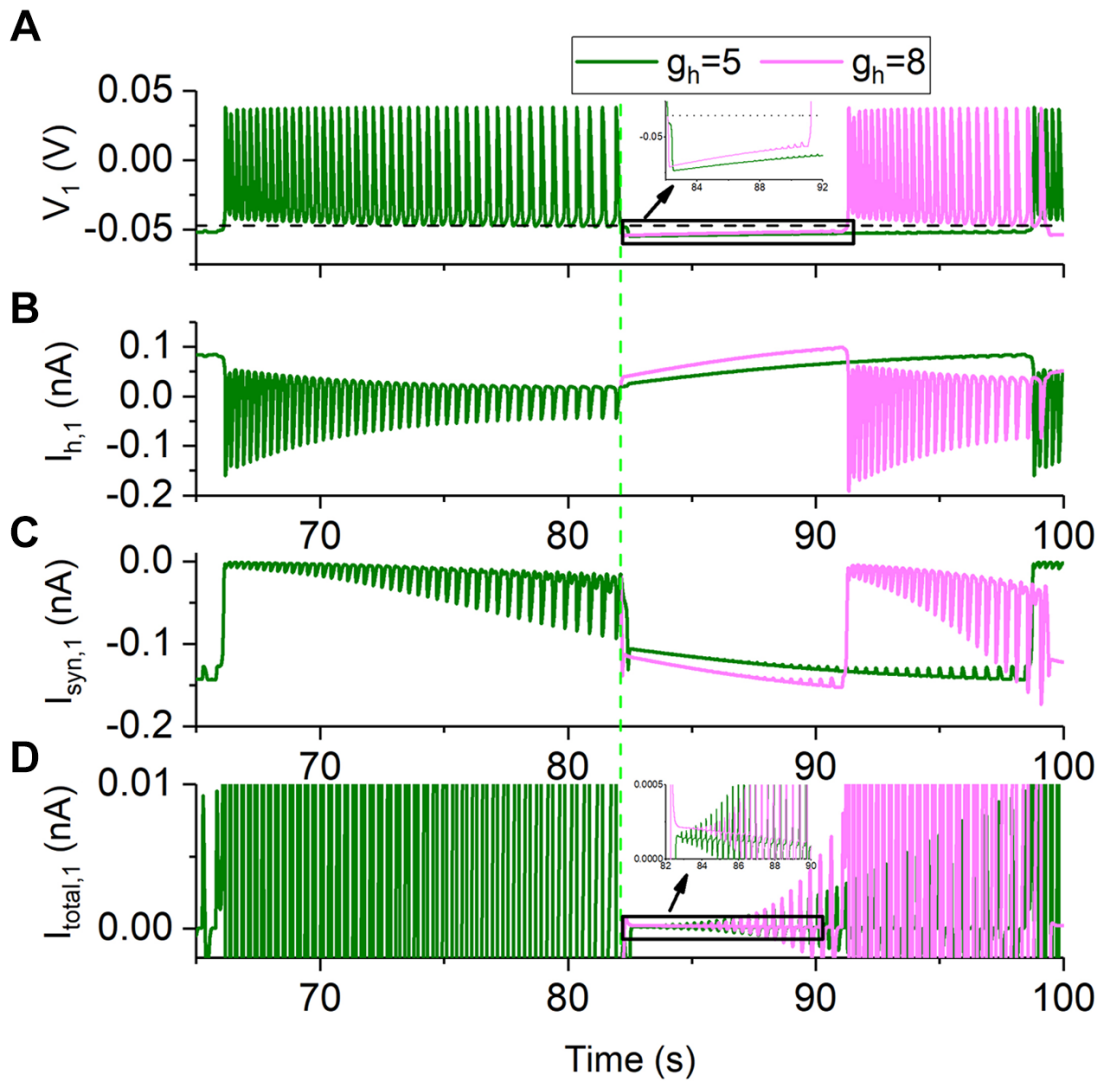
Changes of the membrane potential and ionic currents of the neuron 1 for *g*_syn_ = 15 nS and *V*_th_ = −0.047 V. The bursting at *g*_h_ = 5 nS (olive), and formation process of bursting at *g*_h_ = 8 nS (pink) for time *t* ≥ 82,134 ms. **(A)** Membrane potential *V*_1_. The black horizontal dashed line represents the synaptic threshold voltage *V*_th_ = −0.047 V. **(B)** *I*_h,1_current. **(C)** Inhibitory synaptic current *I*_syn,1_. **(D)** Total current *I*_total,1_. The green dashed vertical line represents *t* = 82,134 ms at which *g*_h_ is changed from 5 to 8 nS.

The roles of the two current in the decreased bursting period with increasing *g*_h_ are addressed in Figure 5, with *g*_syn_ = 15 nS as representative. Here, the neuron 1 is plotted and the neuron 2 is ignored, since the symmetrical behaviors for the two neurons. The behavior for time *t* < 82,134 ms is the bursting with *g*_h_ = 5 nS. Then, *g*_h_ is increased to 8 nS at *t* = 82,134 ms (green dashed vertical line, i.e., the ending point of the burst) and *g*_h_ remains unchanged after *t* = 82,134 ms. The formation process of the antiphase bursting for *g*_h_ = 8 nS begins from *t* = 82,134 ms. Olive curves represent *g*_h_ = 5 nS, and pink curves denote *g*_h_ = 8 nS. The four panels from top to bottom show the membrane voltage *V*_1_, the *I*_h,1_, the *I*_syn,1_, and the total current *I*_total,1_ (i.e., *C*d*V*_1_/d*t*) in turn.

As shown in Figure 5A, the rise rate of the membrane voltage in the silence phase is larger for the stronger *g*_h_. During the silence phase, the larger the *g*_h_, the larger the *I*h current (Figure 5B) and thus the larger the total current. Larger total current induces faster increase of the membrane voltage *V*_1_ (Figure 5A and the insert panel) to be higher than the synaptic threshold *V*_th_ = −0.047 V (the black dashed line in Figure 5A and the insert panel). Then, the inhibitory current from the neuron 1 to the neuron 2 generates, inhibiting the neuron 2 to form the silence and resulting in the earlier appearance of the burst of the neuron 1. Thus, the larger the *g*_h_ is, the shorter the inhibited phase is, showing that the duration of the inhibited phase decreases with increasing *g*_h_, since the period of the bursting is doubled to the duration of the inhibited phase. Then, the period of the bursting decreases with increasing *g*_h_. The result presents a detailed co-regulation process of the *I*_syn_ and *I*_h_ for the escape mode.

Although *I*_syn,1_ received by the neuron 1 becomes stronger with increasing *g*_h_, as shown in Figure 5C, the total current *I*_total,1_ becomes more positive (Figure 5D), induced by more positive *I*_h,1_ (Figure 5B). The results show that the *I*_h_ plays a dominant role, compared with *I*_syn_.

#### 3.2.3 Roles of the two currents for the release mode

**Figure 6:**
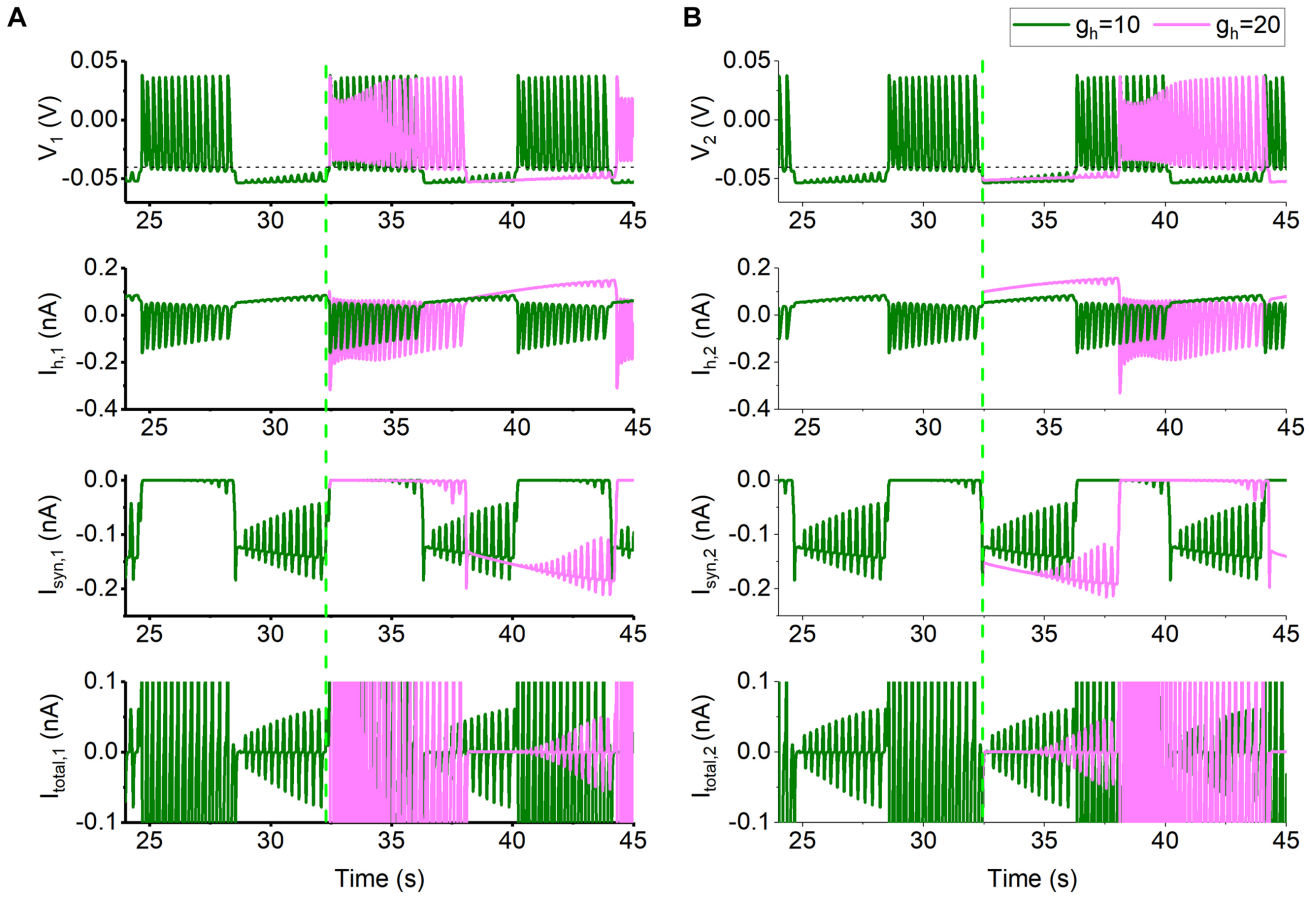
Changes of membrane potential and ionic currents for *g*_syn_ = 15 nS and *V*_th_ = −0.04 V. The bursting at *g*_h_ = 10 nS (olive), and formation process of bursting at *g*_h_ = 20 nS (pink) for *t* > 32,430 ms for neuron 1 shown in **(A)** and for neuron 2 depicted in **(B)**. The panels from top to bottom show the membrane voltage, *I*_h_ current, inhibitory synaptic current *I*_syn_, total current. The black horizontal dashed line represents the synaptic threshold voltage *V*_th_ = −0.04 V. The green dashed vertical line represents *t* = 32,430 ms at which *g*_h_ is changed from 10 nS to 20 nS.

Compared with *V*_th_ = −0.047 V, the *I*_syn_ for *V*_th_ = −0.040 V becomes small. Then, different from *V*_th_ = −0.047 V, the co-regulations of the *I*_syn_ and *I*_h_ for *V*_th_ = −0.040 V can be explained with the burst phase of one neuron and the silence phase of the other neuron. Then, the behaviors of the neurons 1 and 2 are shown in Figure 6. The behavior of the neuron 1 is shown in Figure 6A. The behavior for *t* < 32,430 ms is the antiphase bursting for *g*_h_ = 10 nS and *g*_syn_ = 15 nS. Then, *g*_h_ increases to 20 nS at *t* = 32,430 ms (green dashed vertical line, i.e., the starting point of burst) and *g*_h_ remains unchanged after *t* = 32,430 ms. The behavior of the neuron 2 is shown in Figure 6B. Olive and pink curves appearing after *t* = 32,430 ms represent *g*_h_ = 10 nS and 20 nS, respectively, and the four panels from top to bottom show the membrane voltage *V*, *I*_h_, *I*_syn_, and the total current in turn.

Obviously, the membrane potential, the *I*_h_, and *I*_syn_, and the total current during the silence phase shown in Figure 6A are different from those of *V*_th_ = −0.047 V (Figure 5, the escape mode). Then, the burst of the neuron 1 is chosen as the starting point to explain the co-regulations of the two currents, as shown in Figure 6A. The larger the *g*_h_ is, the larger the *I*_h,1_ current for the neuron 1 is. Then, the larger *I*_h,1_ elevates the valley voltage within the burst (Figure 6A), resulting in a larger synaptic current, *I*_syn,2_, as shown by the pink curve in Figure 6B. The larger *I*_syn,2_ causes the lower membrane voltage in neuron 2, which in turn causes neuron 1 to receive the smaller *I*_syn,1_. Therefore, the time duration in which the valley voltage of the burst is higher than the synaptic threshold *V*_th_ (the black dashed line in Figure 6A) prolongs, resulting in a prolonged burst duration. As the valley voltage within the burst of the neuron 1 falls below *V*_th_ to a certain extent, the inhibitory synaptic current outputted to the neuron 2 (*I*_syn,2_) becomes weak, as shown in Figure 6B, i.e., the maximal negative peak value of the *I*_syn,2_ (pink) after 35 s elevates. As the *I*_syn,2_ becomes small enough to not maintain the silence phase of the neuron 2, the neuron 2 becomes burst and the burst can induce silence of neuron 1 via the inhibitory current *I*_syn,1_, as shown in Figure 6. In a word, the time duration of the valley voltage of the burst of the neuron 1 higher than *V*_th_ increases with increasing *g*_h_, resulting in a prolonged burst duration and bursting period, as shown in Supplementary Figure S3.

### 3.3 Fast-slow dynamics modulated by the *I*_h_ and *I*_syn_ of the antiphase bursting

In the present subsection, the behaviors before and after coupling are analyzed with fast-slow analysis. Firstly, with the one-parameter bifurcations of the fast subsystem of a single neuron, a single neuron is identified to exhibit a spiking behavior, the burst of the antiphase bursting after coupling is related to the stable limit cycle of the fast subsystem, whereas no correspondence to the silence is found. Then, with the two-parameter bifurcations of the fast subsystem of a single neuron, the silence is related to the stable equilibrium point of the fast subsystem to a certain extent. Furthermore, with one-parameter bifurcations of the fast subsystem of the coupled neurons, the silence is related to the unstable equilibrium point. Especially, the roles of the two currents *I*_h_ and *I*_syn_ within the burst and silence phase are discussed.

#### 3.3.1 Fast-slow analysis with one-parameter bifurcations of the fast subsystem of a single neuron

**Figure 7:**
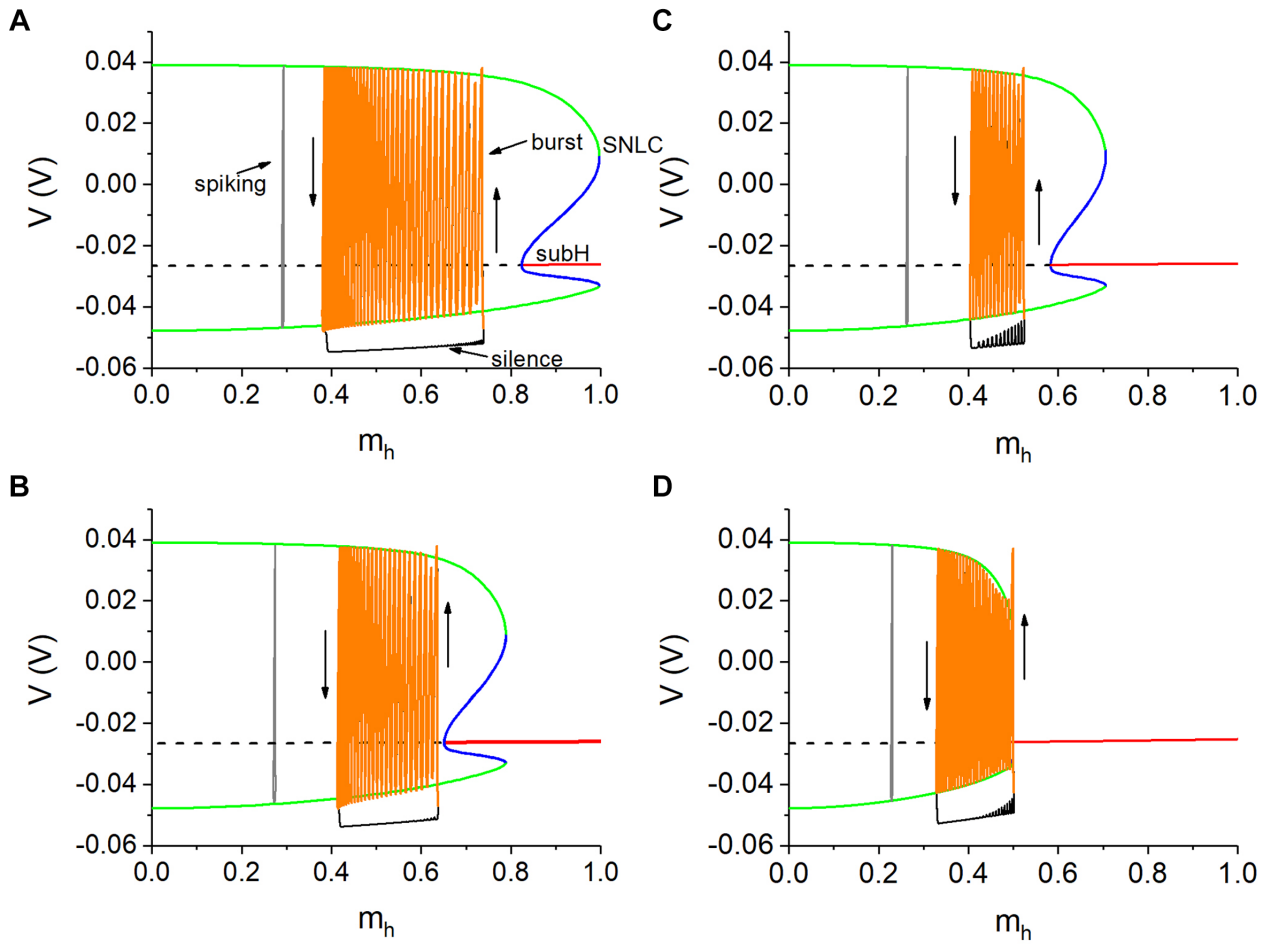
Spikes of burst of the antiphase bursting run along the stable limit cycle of the fast subsystem of the isolated neuron. **(A)** *V*_th_ = −0.047 V and *g*_h_ = 5 nS; **(B)** *V*_th_ = −0.047 V and *g*_h_ = 8 nS; **(C)** *V*_th_ = −0.040 V and *g*_h_ = 10 nS; **(D)** *V*_th_ = −0.040 V and *g*_h_ = 20 nS. Other parameter values: *g*_syn_ = 15 nS and *I*_pol_ = 0.01 nA. Arrow in each panel represents the running direction.

For the single neuron model with the *I*_h_, *m_h_* that described by Equation 4 is the slow variable and Eqs. 1–3 are the fast subsystem. Figure 7 shows the bifurcations of the fast subsystem and trajectory of the spiking for different *g*_h_. In each panel, the unstable equilibrium point (horizontal dashed black line) changes to a stable one (solid red line) via a subcritical Hopf (subH) bifurcation. Meanwhile, an unstable limit cycle (blue curves) emerges, contacting with a stable one (green curves) to form a saddle node bifurcation of the limit cycles (SNLC). Obviously, the spiking (vertical black line in Figure 7) runs along the stable limit cycle and is not related to the stable focus. More details please refer to the Supplementary material and Supplementary Figure S4.

##### 3.3.1.1 Burst of the antiphase bursting is associated with the stable limit cycle

In some previous studies (Gu and Zhao, 2015; Qi et al., 2023), the dynamics of the fast subsystem of an isolated neuron can also characterize the bursting modulated by the synaptic (autaptic) current. Then, the dynamics of the fast subsystem of a single neuron instead of the coupling model is used here. Bifurcations shown in Supplementary Figure S4 are used to characterize the antiphase bursting modulated by the synaptic current, as illustrated in Figure 7. The phase trajectory of the antiphase bursting of the neuron 1 with *I*_pol_ = 0.01 nA is superimposed on Supplementary Figure S4 to form Figure 7, with *V*_th_ = −0.047 V for Figures 7A,B and *V*_th_ = −0.04 V for Figures 7C,D. Obviously, the spikes (orange) of the burst run along the stable limit cycle (green). Within the burst duration of the neuron 1, the inhibitory synaptic current *I*_syn_ from neuron 2 during the silence phase is small or nearly zero, showing that the neuron 1 during the burst resembles an isolated neuron, which is the cause that the burst of the coupled neurons can be explained with the stable limit cycle of the isolated neuron model. Unfortunately, the termination phase of the burst has not been related to a bifurcation, awaiting further studies in future.

##### 3.3.1.2 The *I*_syn_ induces the membrane potential decreased to enhance *m*_h_

Obviously, the bursting appears at *m*_h_ higher than that of the spiking (gray). At the beginning point of the silence phase of the antiphase bursting, the membrane potential is smaller than that of the spiking, induced by the negative *I*_syn_. Then, *m*_h_ at the beginning point becomes larger than that of the spiking, as shown in each panel of Figure 7, since the hyperpolarization activation of the *I*_h_. Then, *m*_h_ during the silence phase increases with respect to time, i.e., from left to right, since *m*_h_ < $m_{h:\infty }\left(m_{h:\infty }=1/\left(1+2e^{180\left(V+\theta_{h}\right)}+e^{500\left(V+\theta_{h}\right)}\right)\right)\text{.}$ Then, $\frac{dm_{h}}{dt}=\frac{m_{h:\infty }-m_{h}}{\tau_{h}}>0$ during the silence phase, resulting in the increase of *m*_h_.

#### 3.3.2 Fast-slow analysis with two-parameter bifurcations of the fast subsystem of a single neuron

**Figure 8:**
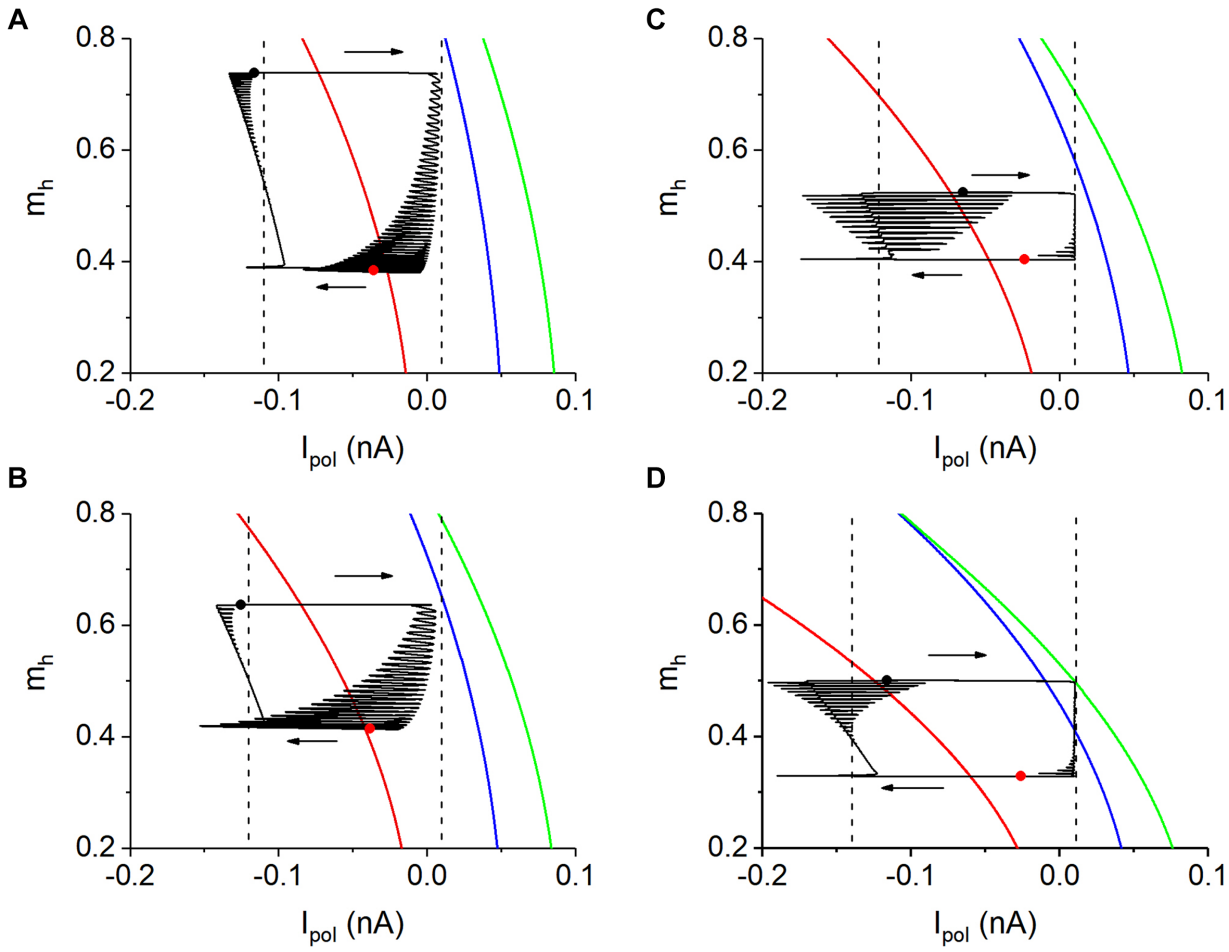
The two-parameter bifurcations and phase trajectory (black curve) in plane (*I*_pol_, *m*_h_). **(A)** *V*_th_ = −0.047 V and *g*_h_ = 5 nS; **(B)** *V*_th_ = −0.047 V and *g*_h_ = 8 nS; **(C)** *V*_th_ = −0.040 V and *g*_h_ = 10 nS; **(D)** *V*_th_ = −0.040 V and *g*_h_ = 20 nS. Parameter: *g*_syn_ = 15 nS. Arrows represent the running direction of bursting. In each panel, phase trajectory from black to red solid circles represents burst, and from red to black solid circles denotes silence phase. Right vertical dashed lines in each panel represents *I*_pol_ = 0.01 nA. Left vertical dashed lines in panels **(A–C)** and **(D)** represent *I*_pol_ = −0.11, −0.12, −0.122, and −0.14 nA, respectively.

As shown in each panel of Figure 7, silence phase shows no relationship to the equilibrium point (red) of the fast subsystem. During the silence phase, the neuron receiving strong *I*_syn_. Then, compared with the isolated neuron, the model of coupling neurons during the silence phase contains a negative *I*_syn_. However, the neuronal model for the bifurcations shown in Figure 7 does not contain an inhibitory coupled current. Then, *I*_pol_ corresponding to *I*_syn_ should be considered. Then, the bifurcations in two-parameter (*I*_pol_, *m*_h_) plane of the fast subsystem of a single neuron are acquired to characterize the antiphase bursting. Here, the bifurcations with respect to *m*_h_ at *I*_pol_ = 0.01 nA (corresponding to zero *I*_syn_) can be used to analyze the burst of the antiphase bursting, as depicted in Figure 8. And the bifurcations with respect to *m*_h_ at a negative *I*_pol_, corresponding to the negative *I*_syn_ during the silence phase, can be used to analyze the dynamics of the silence phase, which are addressed in the following paragraphs.

##### 3.3.2.1 Phase trajectory of the antiphase bursting in the two-parameter plane

Figure 8 show the two-parameter (*I*_pol_ and *m*_h_) bifurcations of the fast subsystem. The green, blue, and red curves represent the saddle-node bifurcation of the limit cycle (SNLC), Hopf bifurcation of the equilibrium point, and saddle-node bifurcation on an invariant circle (SNIC), respectively. With the increase of *I*_pol_ or *m*_h_, the SNIC bifurcation, Hopf bifurcation, and SNLC bifurcation appear. More detailed bifurcations are shown in the Supplementary material and Supplementary Figure S5.

The phase trajectory (*I*_syn_ + 0.01, *m*_h_) of the bursting shown by the black curve and the bifurcations in the plane (*I*_pol_, *m*_h_) are plotted together in each panel of Figure 8. Here, the value “0.01” in “*I*_syn_ + 0.01” is the value of *I*_pol_ for the antiphase bursting. Figures 8A,B correspond to *g*_h_ = 5 nS and *g*_h_ = 8 nS for *V*_th_ = −0.047 V, respectively. Figures 8C,D correspond to *g*_h_ = 10 nS and *g*_h_ = 20 nS for *V*_th_ = −0.04 V, respectively. The bursting trajectory in (*I*_pol_, *m*_h_) plane runs in a clockwise direction, as shown by the arrows in Figure 8, from the black to the red solid circles representing the burst phase, and from the red to the black solid circles denoting the silence phase. The *I*_syn_ changes drastically from the burst to the silence and from the silence to the burst, showing that the *I*_syn_ is related to the alternation between the burst and silence, and *m*_h_ changes drastically within the burst or silence phases, showing that the *I*_h_ current is associated with the dynamics within the two phases.

The silence and burst phases of the antiphase bursting correspond to the low (around left dashed line) and high (close to right dashed line) *I*_syn_ values, respectively. Therefore, the bifurcation of the fast subsystem of the single neuron model with respect to *m*_h_ at a high *I*_pol_ value (*I*_pol_ = 0.01 nA) can be used to explain the burst of the antiphase bursting, which has been addressed in Figure 8. The bifurcations of the fast subsystem with respect to *m*_h_ at a low *I*_pol_ value can be used to explain the silence phase of the antiphase bursting.

##### 3.3.2.2 Silence of the antiphase bursting is related to the stable equilibrium point to a certain extent

**Figure 9:**
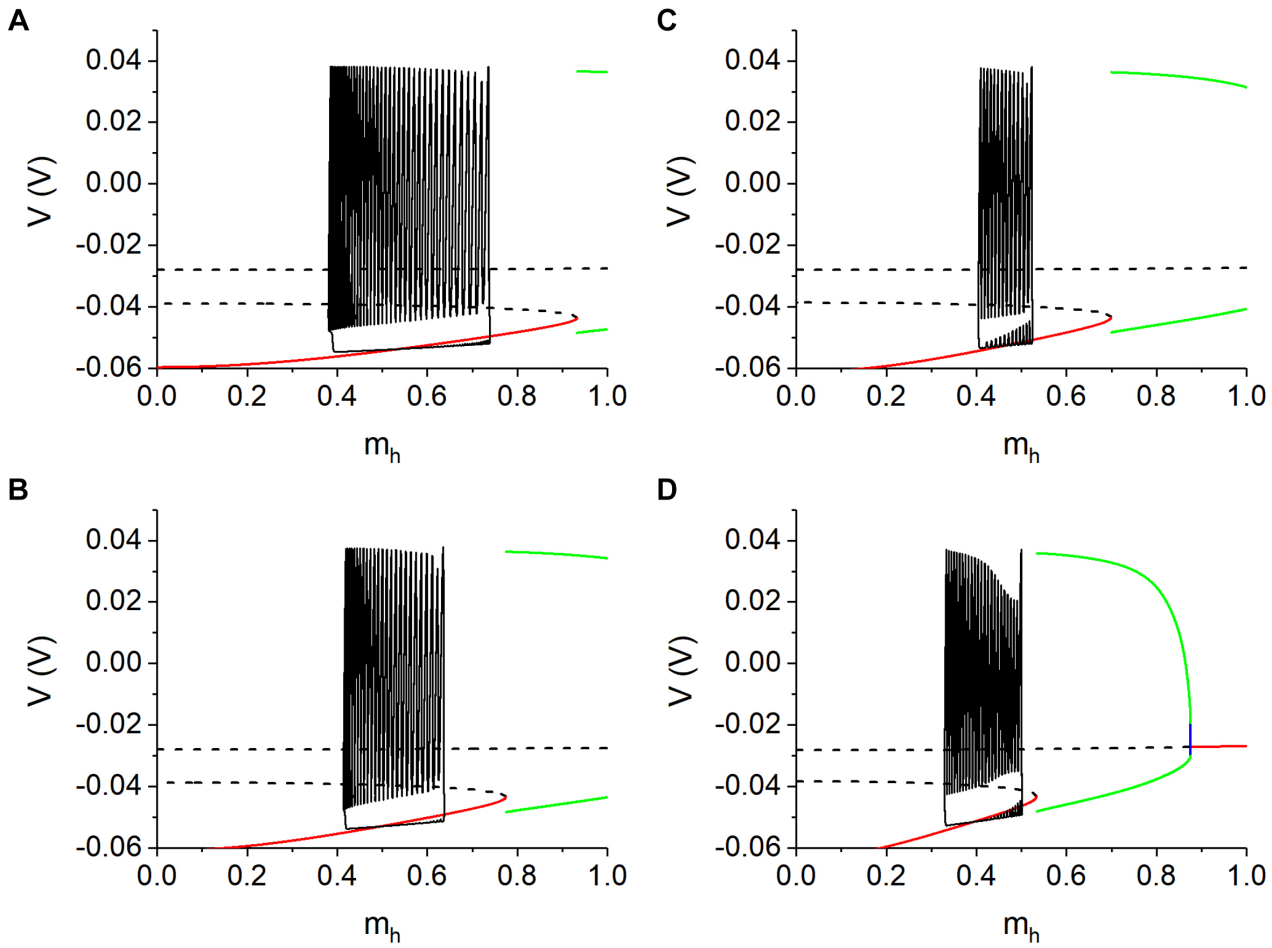
Bursting trajectory (black curve) plotted with bifurcations with respect to *m*_h_ at lower *I*_pol_ values. **(A)** *V*_th_ = −0.047 V, *g*_h_ = 5 nS, and *I*_pol_ = −0.11 nA; **(B)** *V*_th_ = −0.047 V, *g*_h_ = 8 nS, and *I*_pol_ = −0.12 nA; **(C)** *V*_th_ = −0.040 V, *g*_h_ = 10 nS and *I*_pol_ = −0.122 nA; **(D)** *V*_th_ = −0.040 V, *g*_h_ = 20 nS and *I*_pol_ = −0.14 nA. Parameter *g*_syn_ = 15 nS for antiphase bursting.

For *g*_h_ = 5 nS and *V*_th_ = −0.047 V, the bifurcations of the fast subsystem with respect to *m*_h_ at *I*_pol_ = −0.11 nA are shown in Figure 9A, corresponding to those along the left vertical dashed line of Figure 8A. Figures 9B–D show the bifurcations along the left vertical dashed lines of Figures 8B–D respectively. The bifurcations are similar to Supplementary Figure S5D and the parts for larger *m*_h_ are not shown here, due to far away from the bursting trajectory. The phase trajectories of the antiphase bursting of the neuron 1 in the (*m*_h_, *V*) plane for different values of *g*_h_ and *V*_th_ are illustrated by the solid black curves in Figure 9. Each panel shows the silence phase runs near the stable equilibrium point (red curve) of the fast subsystem, showing that the silence phase is associated with the stable equilibrium point to a certain extent. In other words, the silence phase of the antiphase bursting runs around the equilibrium point of the fast subsystem of single neuron, modulated by the changes of *I*_h_ current and negative *I*_syn_ current. The difference between the silence phase and stable equilibrium point (red curve) is induced by the difference between the *I*_syn_ and *I*_pol_.

#### 3.3.3 Fast-slow analysis with bifurcations of the fast subsystem of the coupled neurons

Since the silence behavior of antiphase bursting is not well explained with the single neuron model, fast-slow analysis with bifurcations of the fast subsystem of the coupled neurons are used to analyze the dynamics of the silence behavior in the following paragraphs. For the coupled neurons, the fast subsystem is an eight-dimensional model including Eqs. 5–7, 9 with *i* = 1 and 2. The variables are *V*1, *V*2, *h*_Na,1_, *h*_Na,2_, *m*_K,1_, *m*_K,2_, *S*1 and *S*2, with *m*_h,1_ and *m*_h,2_ taken as bifurcation parameters. Because the exchange of *i* and *j* does not change the model, the fast subsystem also exhibits symmetry. Therefore, *m*_h_ = *m*_h,1_ = *m*_h,2_ is set in the fast subsystem of the coupled neurons. Then, the bifurcations of the fast subsystem in the plane (*m*_h_, *V*) are obtained, which can be used to analyze the dynamics of the antiphase bursting.

##### 3.3.3.1 Bifurcations of the fast subsystem of the coupled neurons

**Figure 10:**
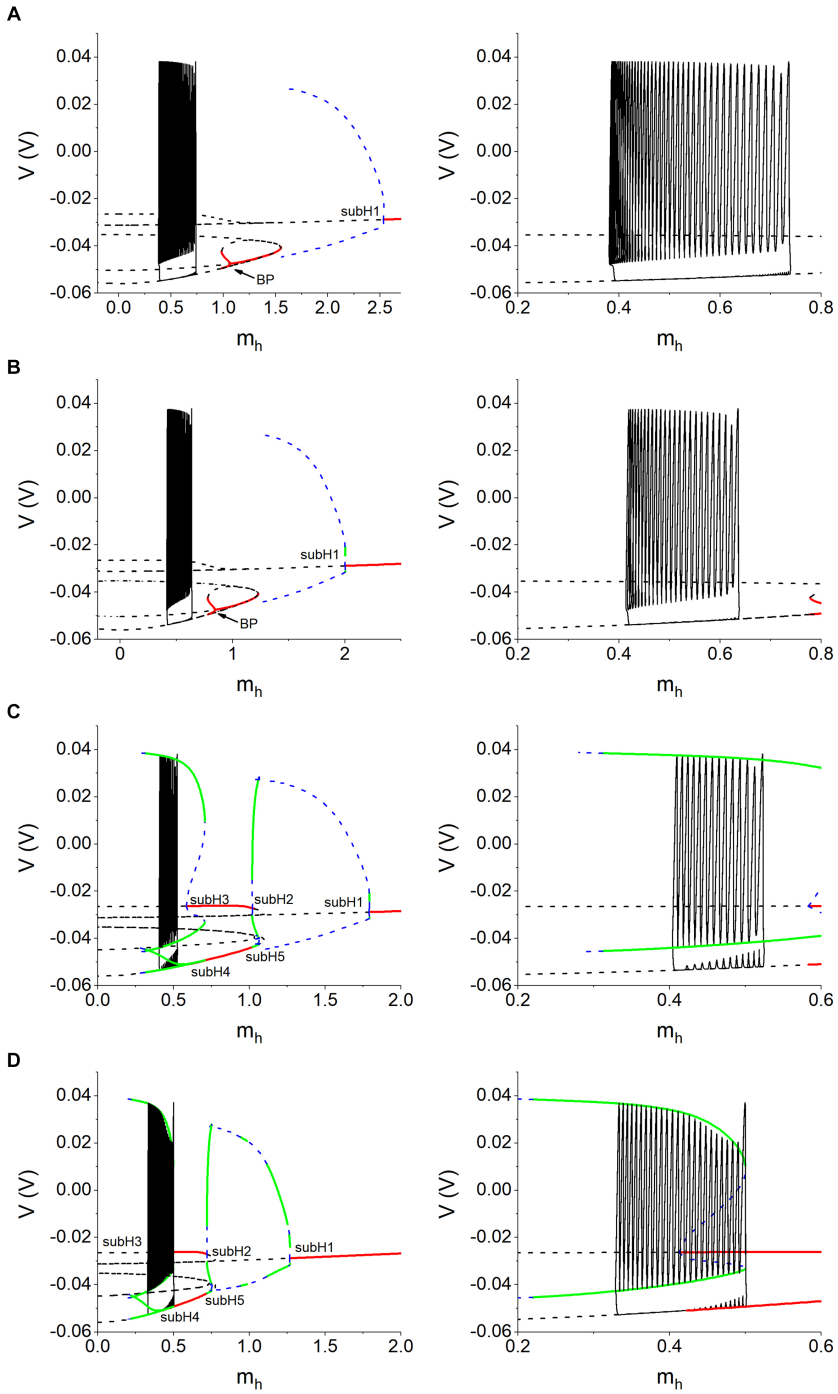
Bursting trajectory (black solid curve) plotted with bifurcations with respect to *m*_h_ of the fast subsystem of the coupled neurons. **(A)** *V*_th_ = −0.047 V and *g*_h_ = 5 nS; **(B)** *V*_th_ = −0.047 V and *g*_h_ = 8 nS; **(C)** *V*_th_ = −0.040 V and *g*_h_ = 10 nS; **(D)** *V*_th_ = −0.040 V and *g*_h_ = 20 nS. Right panels are the partial enlargement of left panels. Other parameter values: *g*_syn_ = 15 nS and *I*_pol_ = 0.01 nA.

The bifurcations of the fast subsystem in the plane (*m*_h_, *V*) are obtained, as shown in the left column of Figure 10. Different rows of Figure 10 represent different combinations of *V*_th_ and *g*_h_. Except for the bursting trajectory (solid black curve), other curves represent the bifurcation curves of the equilibrium points and limit cycles. The solid red curves and dashed black curves represent the stable and unstable equilibrium points, respectively. The dashed blue curves and solid green curves denote the unstable and stable limit cycles, respectively. For *V*_th_ = −0.047 V, the unstable equilibrium point changes into the stable equilibrium point via a subcritical Hopf bifurcation point (label as subH1) at *m*_h_ ≈ 2.53169 nA, and meanwhile an unstable limit cycle appears. At *m*_h_ ≈ 1.55443 nA, the system exhibits a saddle-node bifurcation. At *m*_h_ ≈ 1.06626 nA, there is a branch point (BP), via which the system changes from a stable equilibrium point to two stable equilibrium points and one unstable equilibrium point. The bifurcations for *g*_h_ = 8 nS are similar to the bifurcation for *g*_h_ = 5 nS, which are not described here. The bifurcations for *V*_th_ = −0.04 V are more complex than those for *V*_th_ = −0.047 V, as shown in Figures 10C,D. For *V*_th_ = −0.04 V and *g*_h_ = 10 nS, there are five subcritical Hopf bifurcations at *m*_h_ ≈ 1.79022 (subH1), 1.02003 (subH2), 0.58297 (subH3), 0.70699 (subH4), and 1.01931 (subH5), respectively. An unstable limit cycle bifurcated from the subcritical Hopf bifurcation point is transformed into a stable limit cycle via a saddle-node bifurcation of limit cycles (SNLC) or period-doubling bifurcation (PD), as shown in Figure 10C. Figure 10D shows the bifurcations for *g*_h_  = 20 nS, which are similar to the bifurcations depicted in Figure 10C and will not be repeated here. As shown in Figure 10, there are multiple equilibrium points at a same *m*_h_ value. Furthermore, the bursting trajectory (solid black curve) is plotted with the bifurcations in the plane (*m*_h_, *V*) to present comprehensive and accurate relationships between the antiphase bursting and dynamics of the fast subsystem.

##### 3.3.3.2 Silence of the antiphase bursting is related to the unstable equilibrium point

In order to more clearly show the correspondence between the silence of the antiphase bursting and the equilibrium point of the fast subsystem of the coupled neurons, we omit some irrelevant equilibrium point curves in the bifurcation diagram, leaving only the key equilibrium point curves and limit cycle curves, as shown in the right column of Figure 10. Obviously, in all four panels, the silence of the antiphase bursting runs along the unstable equilibrium point of the fast subsystem, i.e., the silence of the antiphase bursting is related to the unstable equilibrium point. The result indicates that the neuron is at the unstable equilibrium point during the silence phase suppressed by the inhibitory synaptic current. Supplementary Figure S6 shows a comparison of the stable equilibrium point of the fast subsystem of a single neuron and the unstable equilibrium point of the fast subsystem of the coupled neurons.

In addition, other complex dynamics can be found from Figure 10. For example, for *V*_th_ = −0.040 V, the silence of the antiphase bursting runs along the unstable equilibrium point of the fast subsystem, and the burst runs along the stable limit cycle, as shown in Figures 10C,D. For *V*_th_ = −0.040 V and *g*_h_ = 10 nS, the subcritical Hopf (subH1) bifurcation occurs at *m*_h_ ≈ 0.58297. For *V*_th_ = −0.040 V and *g*_h_ = 20 nS, the subcritical Hopf (subH1) bifurcation appears at *m*_h_ ≈ 0.41215, i.e., the Hopf bifurcation point shift to the left with the increase of *g*_h_. For *V*_th_ = −0.047 V, the Hopf bifurcation disappears, as shown in Figures 10A,B. These complex bifurcations will be studied in details in future.

## 4 Discussion and conclusion

The rhythmic patterns of two-neuron circuit with reciprocal inhibition coupling, such as the antiphase bursting, are associated with complex nonlinear dynamics and motor patterns (Daun et al., 2009; Nagornov et al., 2016; Elices and Varona, 2017; Ausborn et al., 2018). Especially, the rhythmic patterns have been widely used to control motion of the robot in recent studies (Habu et al., 2019; Li J. et al., 2021; Fukuoka et al., 2022). Identifying modulations to the rhythmic patterns of two-neuron circuit with reciprocal inhibition coupling is a very important issue for the nonlinear dynamics, neuroscience, and motion control of the robot. In the present paper, relationships among the antiphase bursting and multiple other rhythm patterns, and the dependence of the antiphase bursting on the *I*_h_ and *I*_syn_ at different values of *V*_th_, the dependence of the escape, release, and mixture modes of the antiphase bursting on the *I*_h_ and *I*_syn_, co-regulations of the *I*_h_ and *I*_syn_ in modulating the opposite changes of the bursting period of the escape mode and release mode, and fast-slow dynamics modulated by the *I*_h_ and *I*_syn_ of the antiphase bursting, are investigated via different processes of fast-slow dissection methods in a two-neuron model (conductance-based leech neuronal model). The results exhibit significances in the following aspects.

Firstly, the *I*_syn_ and the slow *I*_h_ with strong strength are the necessary conditions for the antiphase bursting, and the dependence of the antiphase bursting on the two currents is different for low (escape mode) and high (releases mode) threshold voltages of the inhibitory synapse. Multiple rhythmic patterns are reproduced in the model with or without the *I*_h_ current, and the relationships between different patterns are acquired. The antiphase bursting pattern, which locates between the antiphase spiking and the pattern of spiking and subthreshold oscillation, are induced by the *I*_h_. The slow activation of the *I*_h_ is identified to be the factor to ensure the generation of bursting, which presents explanation to the experimental observation that the antiphase bursting appears in the presence of *I*_h_ current (Sharp et al., 1996). Meanwhile, as the synaptic threshold voltage shifts from low level to high level, the dependence of the antiphase bursting on the *I*_syn_ and *I*_h_ is different, suggesting that the interaction of the strong *I*_h_ and *I*_syn_ induces the antiphase bursting.

Secondly, with increasing *I*_h_, the bursting period decreases and increases, respectively, for the escape mode and release mode, which are reproduced in the simulations, closely matching the experimental observation (Sharp et al., 1996; Morozova et al., 2022). In addition, the mixture mode observed in Morozova et al. (2022) is also reproduced at a medium synaptic threshold voltage. Furthermore, the co-regulations of the *I*_h_ and *I*_syn_ to modulate the opposite changes of the bursting period for the escape mode and release mode are obtained. For the escape mode, increase of the *I*_h_ induces elevated membrane potential of the silence inhibited by a strong *I*_syn_, which reduces the silence duration to go beyond *V*_th_, resulting in reduced bursting period. For the release mode, the *I*_h_ elevates the tough value of the former part of the burst of the neuron 1 modulated by a nearly zero *I*_syn,1_, meanwhile, an enhanced *I*_syn,2_ is outputted to the silence of the neuron 2 to prolong the silence duration. As the tough value of the burst of the neuron 1 falls below *V*_th_, the *I*_syn,2_ outputted to the neuron 2 decrease to nearly zero and then the neuron 2 changes to burst. The burst of the neuron 2 induces strong *I*_syn,1_ to the neuron 1 to terminate the burst to become the silence. Then, the co-regulations of the two currents for the release mode are more complex than those the escape mode in the present paper and previous study. In addition, the co-regulations of the two currents for the release mode seems much clearer than those in Sharp et al. (1996), wherein the tough value of the burst does not fall below *V*_th_. In this paper, the detailed dependence of the bursting period on two key factors (the *I*_h_ and *I*_syn_) for the escape mode and release mode is obtained in a two-parameter plane. Then, through modulations to the two physiological factors, antiphase bursting patterns with various periods can be obtained, which may be applied as potential and practical measures to modulate the motion of robot.

Finally, the fast-slow dynamics of the antiphase bursting modulated by the *I*_h_ and *I*_syn_ are acquired. Using one-parameter bifurcations of the fast subsystem of a single neuron, the burst of the antiphase bursting is related to the stable limit cycle modulated by a weak *I*_syn_, and the silence is not associated the dynamics of the fast subsystem. Considering that the antiphase bursting is modulated by the inhibitory synaptic current (nearly zero during the burst and negative during the silence phase) and the slow *I*_h_, fast-slow analysis considering two bifurcation parameters related to the two currents are used in the present paper. Such a novel analysis process is verified to be effective to analyze the dynamics of the antiphase bursting and roles of the *I*_syn_ and *I*_h_ in the antiphase bursting. The bifurcations of the fast subsystem at a high depolarization current show that the burst is related to the stable limit cycle corresponding to a weak inhibitory synaptic current, while the silence is associated with the stable equilibrium point to a strong inhibitory synaptic current to a certain extent. Although the silence is close to the stable equilibrium, the silence exhibits difference to the stable equilibrium. Furthermore, the dynamics of the fast subsystem of the coupled neurons is used, then, the silence is associated with the unstable equilibrium point. The *I*_syn_ induces the alternation between the burst and the silence, and the *I*_h_ mainly modulates the dynamics within the burst and quiescent state. The fast-slow analysis considering two-parameter bifurcations or coupled system are novel progresses used in recent years (Li Y. et al., 2021; Ma et al., 2021). The results present theoretical explanations to the fast-slow dynamics of the antiphase bursting modulated by the *I*_syn_ and *I*_h_, which is helpful for understanding the antiphase bursting and modulating the rhythmic motor behavior.

Although the progresses in the three aspects mentioned above, there are multiple questions related to the mechanism and co-regulations of the *I*_h_ and *I*_syn_ to be answered in future. For example, the co-regulations of the two currents in the formation of the mixture mode of antiphase bursting await further investigations. Moreover, the dynamical mechanisms by which other currents interact with the *I*_h_ and *I*_syn_ to regulate the antiphase bursting are also questions to be answered in the future. More importantly, the generality of the results of the present paper should be verified with multiple theoretical models and the heterogeneous parameter values, closely matching the real neurons or systems. Especially, the bifurcations undying the alternation between burst and silence have not been acquired with fast-slow analysis considering one or two bifurcation parameters, implying that some novel analysis process may be needed. The study to these problems can enable us to further understand the mechanisms of different rhythm patterns more deeply and comprehensively, which is helpful for the application the antiphase bursting in motion control of robot and so on. In addition, the *I*_h_ is a special current which can modulate the threshold (Guan et al., 2019) and resonances (Guan et al., 2020, 2021) and is associated with the dendritic integration, synaptic transmission, motor learning, pacemaker function, and pathologies (Robinson and Siegelbaum, 2003; Biel et al., 2009; Wahl-Schott and Biel, 2009), which should be further studied in future.

## Data availability statement

The raw data supporting the conclusions of this article will be made available by the authors, without undue reservation.

## Ethics statement

Ethical approval was not required for the study involving animals in accordance with the local legislation and institutional requirements because no real animals were involved in this study. This study presents the simulation and analysis results of a theoretical model.

## Author contributions

LG: Data curation, Investigation, Software, Writing – original draft, Validation, Visualization, Writing – review & editing. HG: Conceptualization, Methodology, Supervision, Writing – review & editing, Visualization, Writing – original draft. XZ: Formal analysis, Methodology, Writing – review & editing, Validation.
