# Supplementary material for: Dynamics of antiphase bursting modulated by the inhibitory synaptic and hyperpolarization-activated cation currents
Source: Front Comput Neurosci. 2024 Feb 9;18:1303925. doi: 10.3389/fncom.2024.1303925 (PMC10884300; doi:10.3389/fncom.2024.1303925)
Supplement: Supplementary file 2 [file Data_Sheet_2.DOCX]

Supplementary Material

# Activation functions of the *I*_h_ and the inhibitory synapse

To clearly study the roles of the *I*_syn_ and *I*_h_ in the antiphase bursting, the activation functions of the two currents are shown in supplementary Fig. 1. The green curve represents the activation curve of the *I*_h_, i.e., $m_{h\_\infty}=1/{[1+2e^{180\left( V+\theta_{h} \right)}+e^{500\left( V+\theta_{h} \right)}]}$ with *θ*_h_ = −0.04 V. The lower the membrane voltage *V* is, the larger the $m_{h\_\infty}$ is, showing the hyperpolarization characteristic of the *I*_h_. The blue and red curves (Fig. 6) represent the activation curves of the inhibitory synapse $S_{\infty}\left( V \right)=1/[1+e^{-1000\left( V-V_{\mathrm{th}} \right)}]$ for *V*_th_ = −0.047 V and *V*_th_ = −0.04 V, respectively. At a same membrane potential *V*, the lower the *V*_th_ is, the larger the $S_{\infty}$ is, and the larger the corresponding inhibitory synaptic current is.


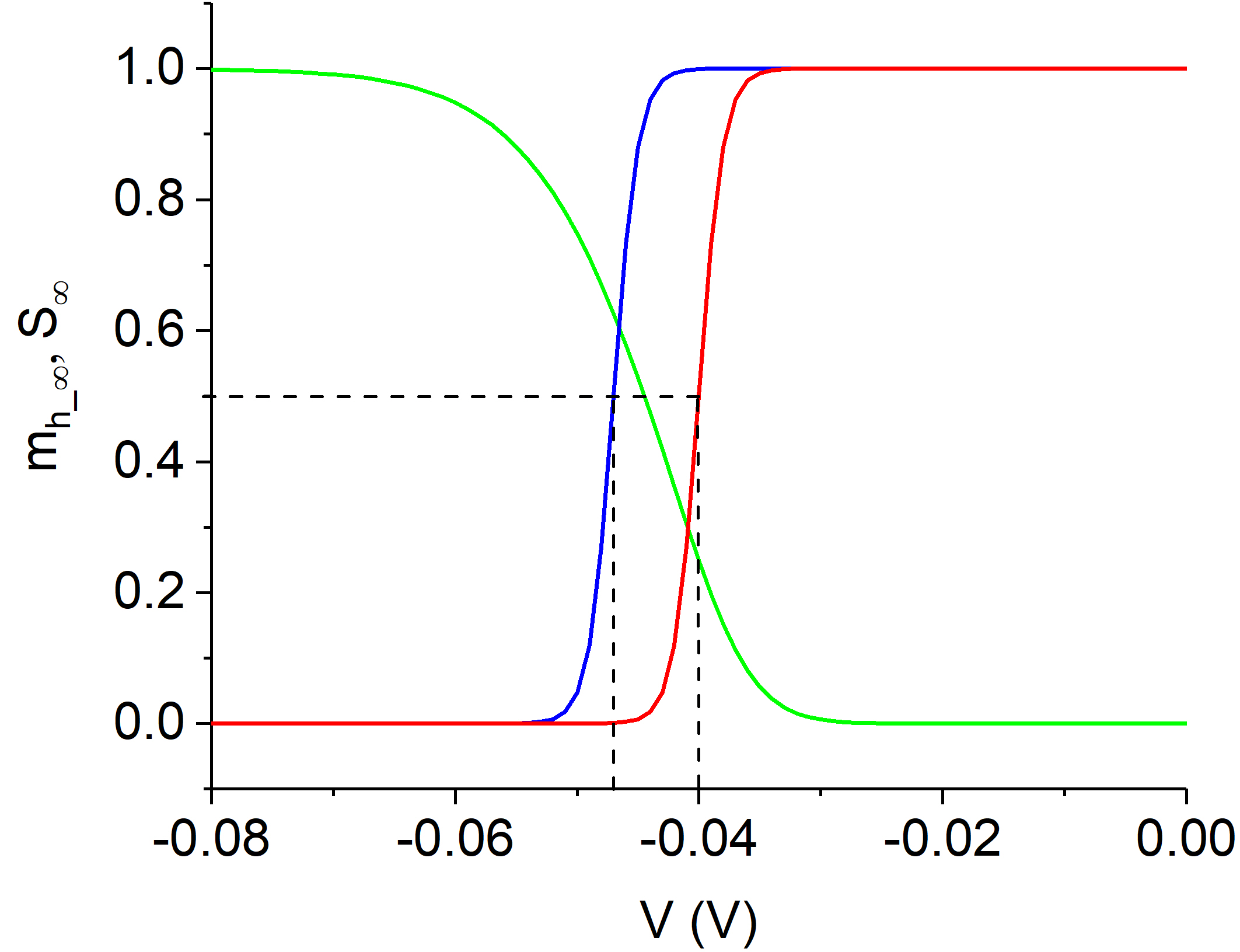


**Supplementary Figure 1.** Activation curves of the *I*_h_ (green) and *I*_syn_ (blue and red for *V*_th_ = −0.047 V and −0.04 V, respectively).

# Opposite changes of the bursting period for the escape mode and release mode

For *V*_th_ = −0.047 V (escape mode), the period of antiphase bursting decreases with the increase of *g*_h_. The bursting period is 32.64, 16.14, and 8.72 s for *g*_h_ = 5, 8, and 12 nS, respectively, as illustrated in supplementary Fig. 2(a). On the contrary, for *V*_th_ = −0.04 V (release mode), the period increases with the increase of *g*_h_. The bursting period is 4.00, 7.78, and 10.61 s for *g*_h_ = 6, 10, and 20 nS, respectively, as shown in supplementary Fig. 2(b). It shows that the change of the bursting period is different for the escape mode and release mode. With increasing *g*_h_, the bursting period decreases for a lower *V*_th_ (escape mode) and increases for a higher *V*_th_ (release mode). For *V*_th_ = −0.047 V, the period decreases for *g*_syn_ = 12 (black), 15 (red), and 18 nS (green), as depicted in supplementary Fig. 2(c). For *V*_th_ = −0.040 V, the period increases for *g*_syn_ = 10 (black), 15 (red), and 20 nS (green), as illustrated in supplementary Fig. 2(d). The results are consistent with the experimental results in Refs [34, 45].


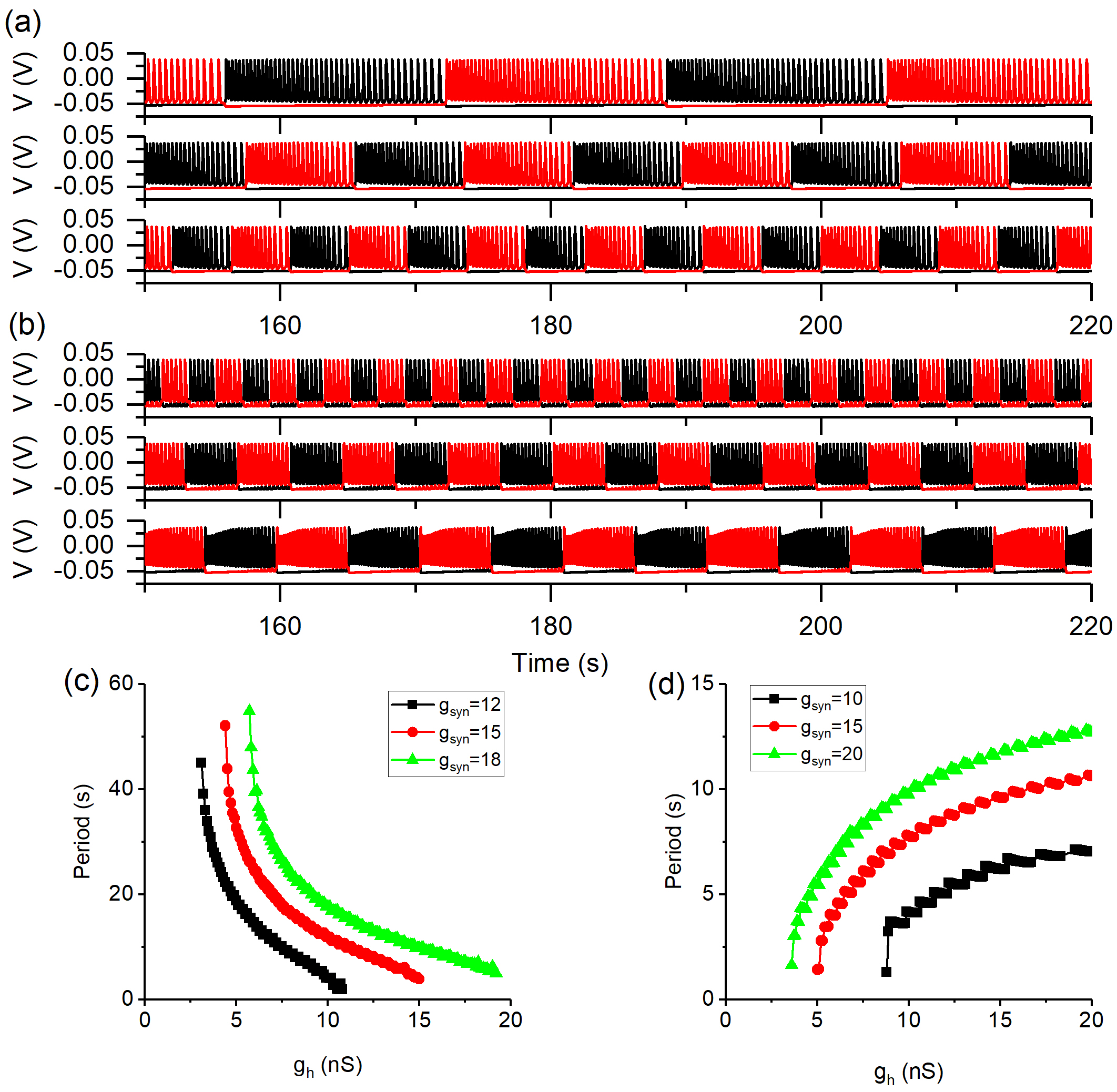


**Supplementary Figure 2.** Changes of the period of the antiphase bursting for different *V*_th_ values. (a) *V*_th_ = −0.047 V and *g*_syn_ = 15 nS. Upper, middle, and lower panels represent *g*_h_ = 5, 8, and 12 nS, respectively; (b) *V*_th_ = −0.04 V and *g*_syn_ = 15 nS. Upper, middle, and lower panels denote *g*_h_ = 6, 10, and *g*_h_ = 20 nS. *g*_syn_ = 15 nS; (c) *V*_th_ = −0.047 V. Changes with respect to *g*_h_ for *g*_syn_ = 12 (black), 15 (red), and 18 (green) nS; (d) *V*_th_ = −0.040 V. Changes with respect to *g*_h_ for *g*_syn_ = 10 (black), 15 (red), and 20 (green) nS.

# Changes of burst for different *g*_h_ value in release mode

The results mentioned in subsection 3.2.3 can also be illustrated by the changes of the burst for *g*_h_ = 10 nS (olive) and *g*_h_ = 20 nS (pink) in supplementary Fig. 3, with the termination points of the burst plotted at a same time. Obviously, for a larger *g*_h_, the time duration of the valley voltage of the burst *V*_1_ higher than the threshold (dashed horizontal line) becomes longer, appearing at the beginning part of the burst, which is induced by the stronger *I*_h,1_ depicted in the middle panel of supplementary Fig. 3 and the weaker *I*_syn,1_ illustrated in the bottom panel of supplementary Fig. 3. These results present a more detailed co-regulation process of the *I*_syn_ and *I*_h_ for the release mode of antiphase bursting^[34]^.


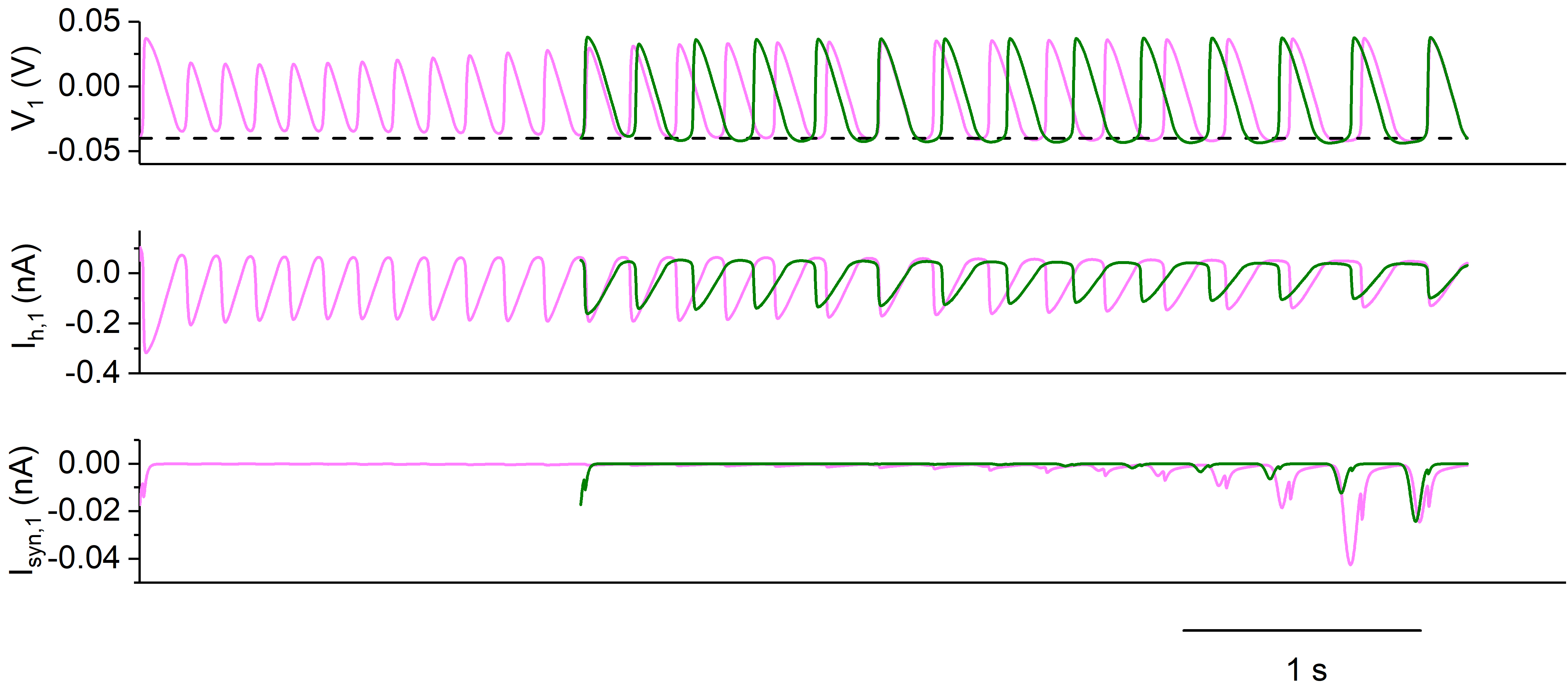


**Supplementary Figure 3.** Changes of burst for *g*_h_ = 10 nS (olive) and *g*_h_ = 20 nS (pink) with the termination points of the burst plotted at a same time. The panels from top to bottom are membrane potential, *I*_h_ current, and inhibitory synaptic current for neuron 1. Other parameters: *g*_syn_ = 15 nS, *V*_th_ = −0.04 V.

# **Spiking of isolated neuron is related to the stable limit cycle**

Supplementary Fig. 4(a), (b), (c), and (d) show the bifurcations of the fast subsystem and trajectory of the spiking for *g*_h_ = 5, 8, 10, and 20 nS, respectively. In each panel, the unstable equilibrium point (horizontal dashed black line) changes to a stable one (solid red line) via a subcritical Hopf (subH) bifurcation. Meanwhile, an unstable limit cycle (blue curves) emerges, contacting with a stable one (green curves) to form a saddle node bifurcation of the limit cycles (SNLC). The solid black vertical curves represent the phase trajectory of spiking of the single neuron for *I*_pol_ = 0.01 nA. The bifurcation points appear at different locations in different panels. Obviously, in each panel, the spiking (vertical black line) runs along the stable limit cycle and is not related to the stable focus. The insert panel in each panel shows the enlargement of the spiking along *m*_h_ direction.


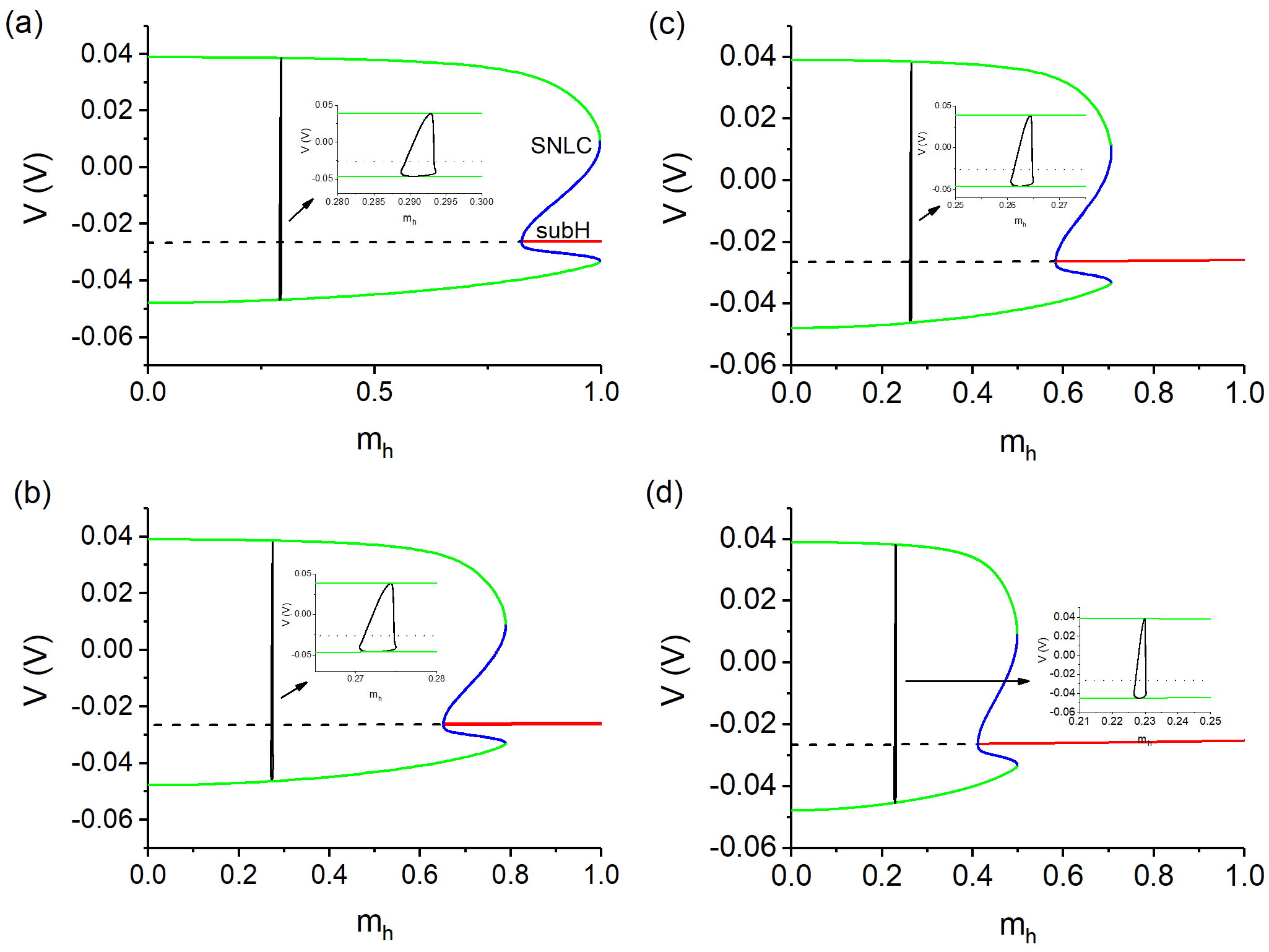


**Supplementary Figure 4.** Bifurcations of the fast subsystem and phase trajectory of the spiking of a single neuron. (a) *g*_h_ = 5 nS; (b) *g*_h_ = 8 nS; (c) *g*_h_ = 10 nS; (d) *g*_h_ = 20 nS. Horizontal dashed black curve and solid red curve represent the unstable and stable equilibrium points, respectively, blue and green curves represent the unstable and stable limit cycles, respectively, solid black vertical curve denotes the phase trajectory of the spiking for *I*_pol_ = 0.01 nA. The insert is a partial amplification of the spiking along *m*_h_ direction.

# **Two-parameter bifurcations**

Supplementary Fig. 5(a) and (b) show the two-parameter (*I*_pol_ and *m*_h_) bifurcations of the fast subsystem. The green, blue, and red curves represent the saddle-node bifurcation of the limit cycle (SNLC), Hopf bifurcation of the equilibrium point, and saddle-node bifurcation on an invariant circle (SNIC), respectively. Supplementary Fig. 5(b) shows the results of supplementary Fig. 5(a) for *m*_h_ > 0. With the increase of *I*_pol_ or *m*_h_, the SNIC bifurcation, Hopf bifurcation, and SNLC bifurcation appear.


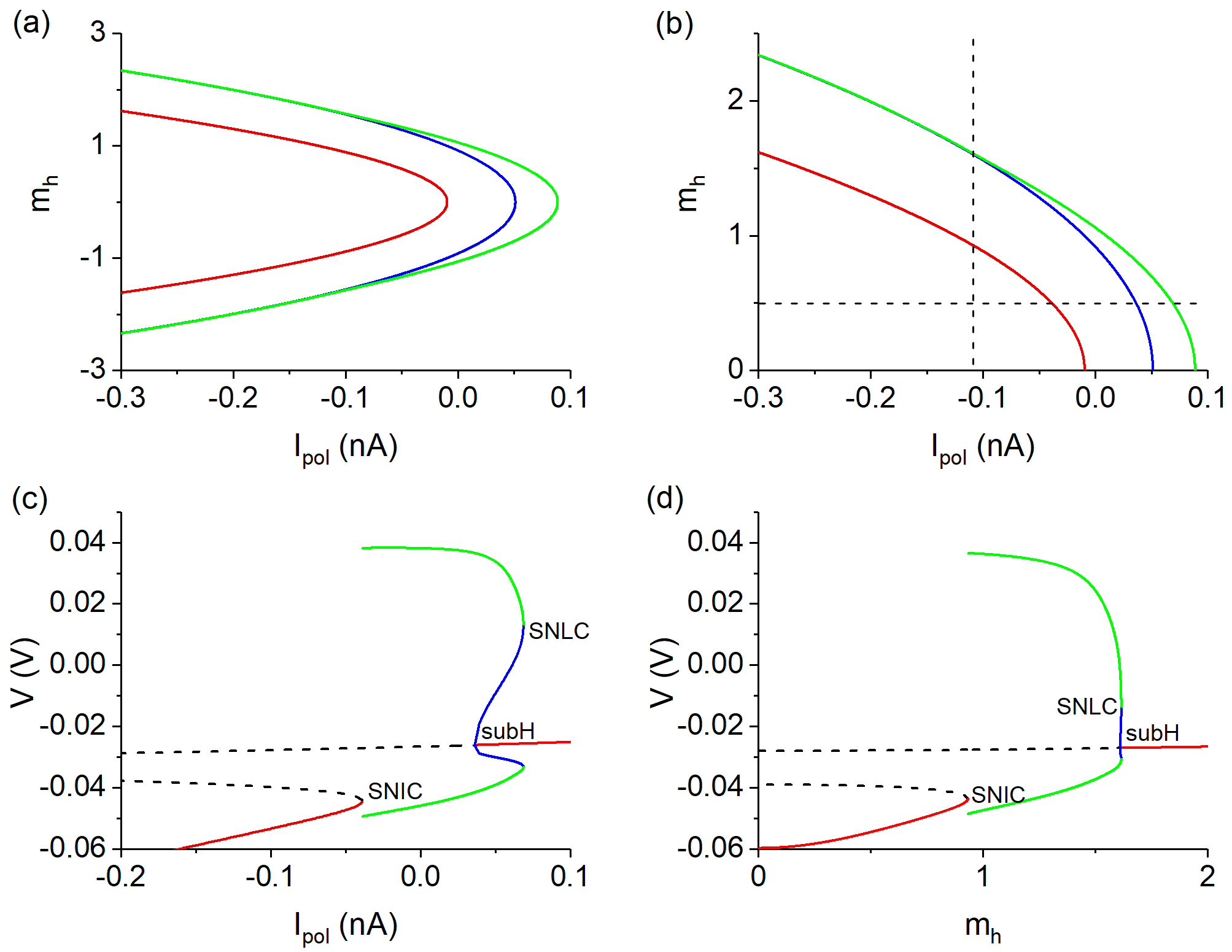


**Supplementary Figure 5.** Bifurcations of the fast subsystem for *g*_h_ = 5 nS. (a) Two-parameter (*I*_pol_, *m*_h_) bifurcations; (b) Local enlargement of panel (a). Horizontal and vertical dashed lines represent *m*_h_ = 0.5 and *I*_pol_ = −0.11 nA, respectively; (c) Bifurcations with respect to *I*_pol_ at *m*_h_ = 0.5; (d) Bifurcation with respect to *m*_h_ at *I*_pol_ = −0.11 nA.

The bifurcations with respect to *I*_pol_ at *m*_h_ = 0.5 and with respect to *m*_h_ at *I*_pol_ = −0.11 nA are illustrated in supplementary Fig. 5(c) and (d), respectively. The red solid curve and black dashed curve represent the stable and unstable equilibrium points, respectively. The green and blue curves denote the stable and unstable limit cycles, respectively. With increasing *I*_pol_ or *m*_h_, the stable equilibrium point and the unstable equilibrium point collide to form the SNIC bifurcation and meanwhile the stable limit cycle appears. For the relatively high membrane voltage, the unstable equilibrium transits to the stable equilibrium point via the subcritical Hopf (subH) bifurcation point, and an unstable limit cycle (blue curve) appears. The unstable and stable limit cycle collide to form the SNLC bifurcation.

# **Difference between the stable equilibrium point of the fast subsystem of a single neuron and the unstable equilibrium point of the fast subsystem of the coupled neurons**

For *V*_th_ = −0.040 V and *g*_h_ = 10 nS, the bifurcations of the fast subsystem of a single neuron and the coupled neurons are compared, as shown in supplementary Fig. 6, with the solid black curve to represent the phase trajectory of the bursting. The solid pink curve and dashed orange curves represent the stable and unstable equilibrium points of the fast subsystem of a single neuron model, respectively. The solid red curves and dashed black curves denote the stable and unstable equilibrium points of the fast subsystem of the coupled neuron model, respectively. The equilibrium points of the two fast subsystems are significantly different, since the two fast subsystems are different. The fast subsystem of the coupled neurons contains the inhibitory coupling current with oscillations, while the fast subsystem of a single neuron contains a constant current to simulate the coupling current. Then, the silence phase of the bursting runs along the equilibrium point of the coupled system.

~~
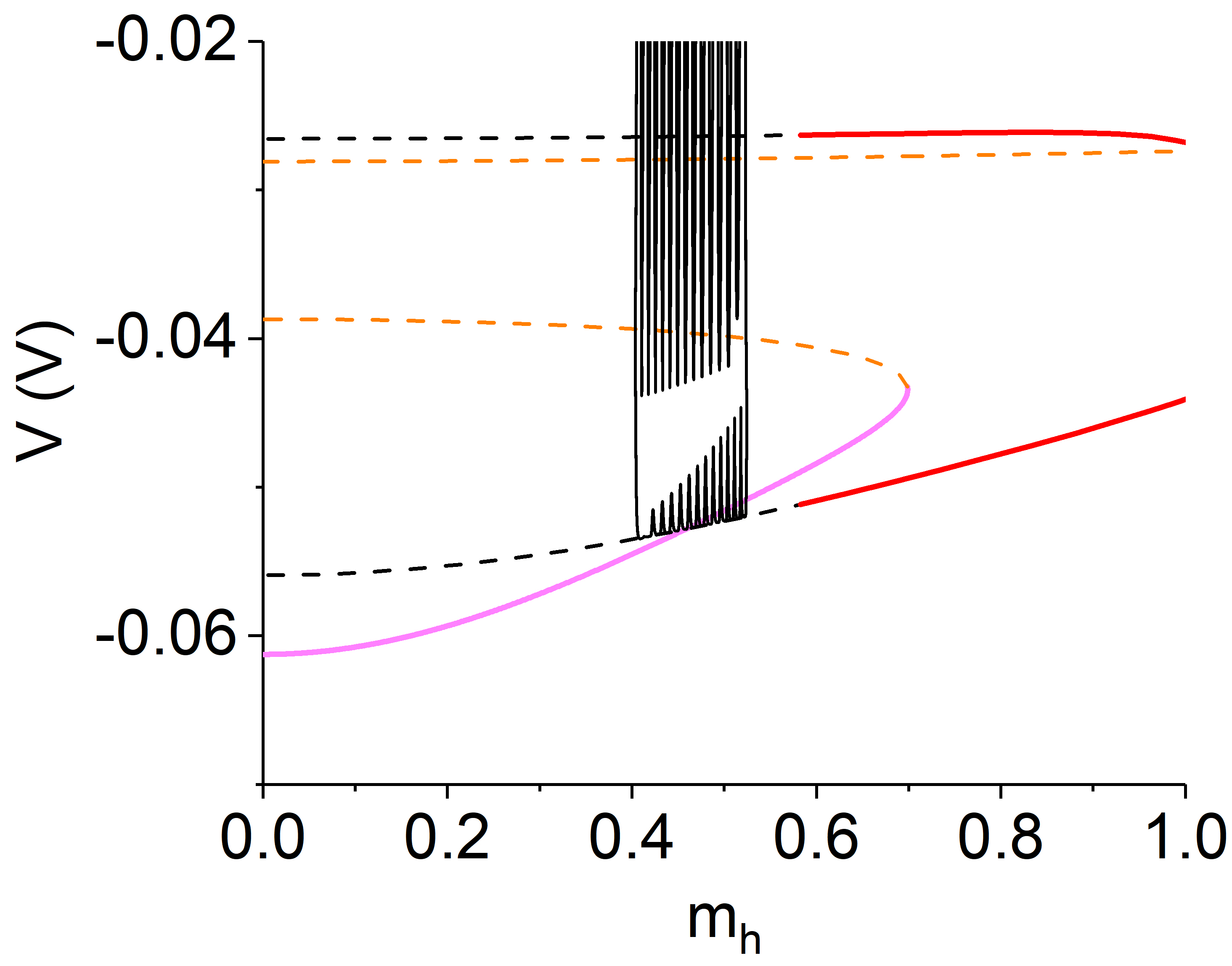
~~

**Supplementary Figure 6.** Bifurcations related to silence phase of the fast subsystem of the coupled neurons (solid red curve and dashed black curves) and a single neuron (solid pink curve and dashed orange curves), plotted with the phase trajectory of the antiphase bursting (black solid curve). Coupled neurons: *V*_th_ = −0.04 V, *g*_syn_ = 15 nS, *g*_h_ = 10 nS, and *I*_pol_ = 0.01 nA. Single neuron: *g*_h_ = 10 nS and *I*_pol_ = −0.122 nA.
