# Supplementary figures and images for: Dynamics of antiphase bursting modulated by the inhibitory synaptic and hyperpolarization-activated cation currents
Source: Front Comput Neurosci. 2024 Feb 9;18:1303925. doi: 10.3389/fncom.2024.1303925 (PMC10884300; doi:10.3389/fncom.2024.1303925)

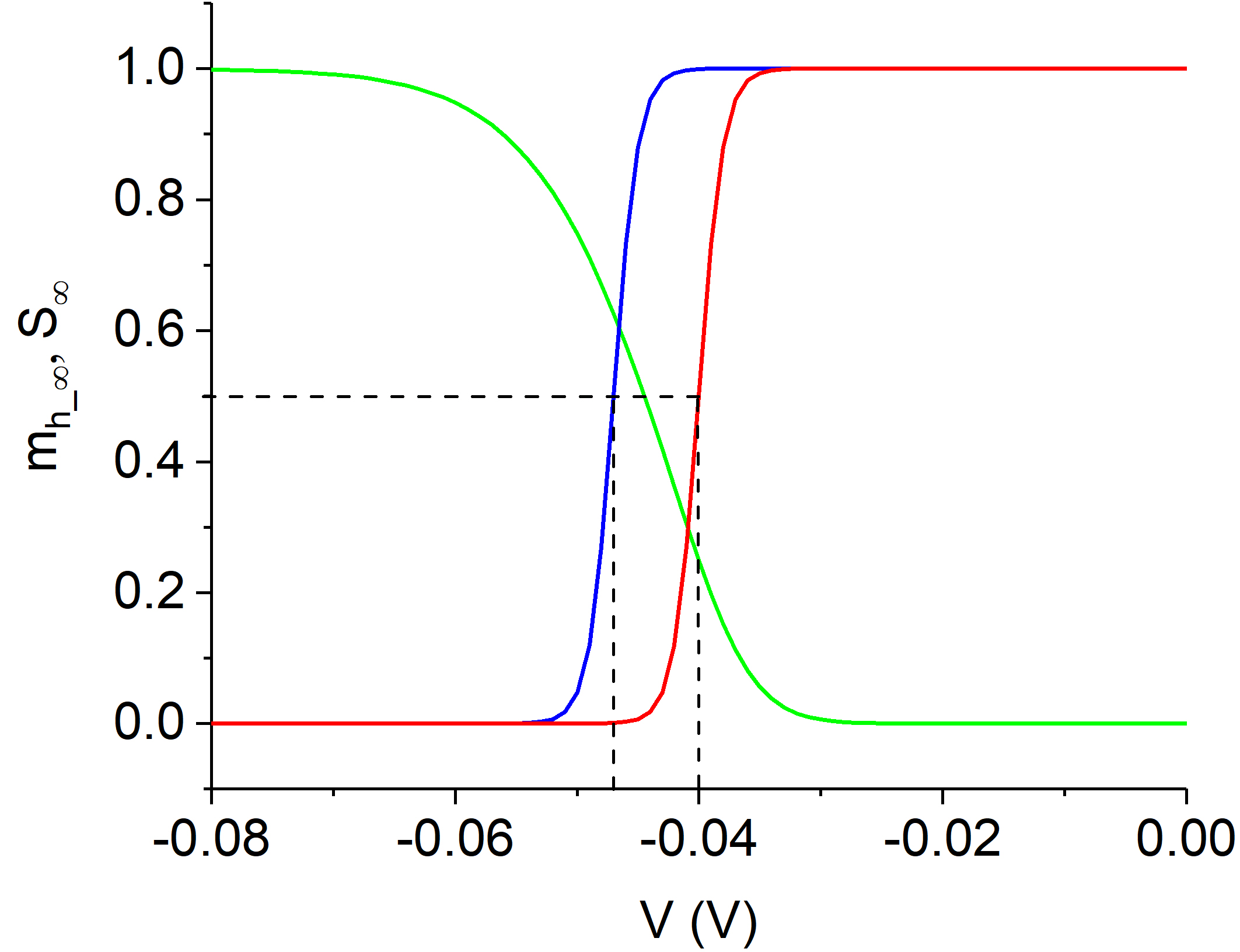

Supplement: Supplementary file 3 [file Data_Sheet_3.ZIP › S1.jpg]

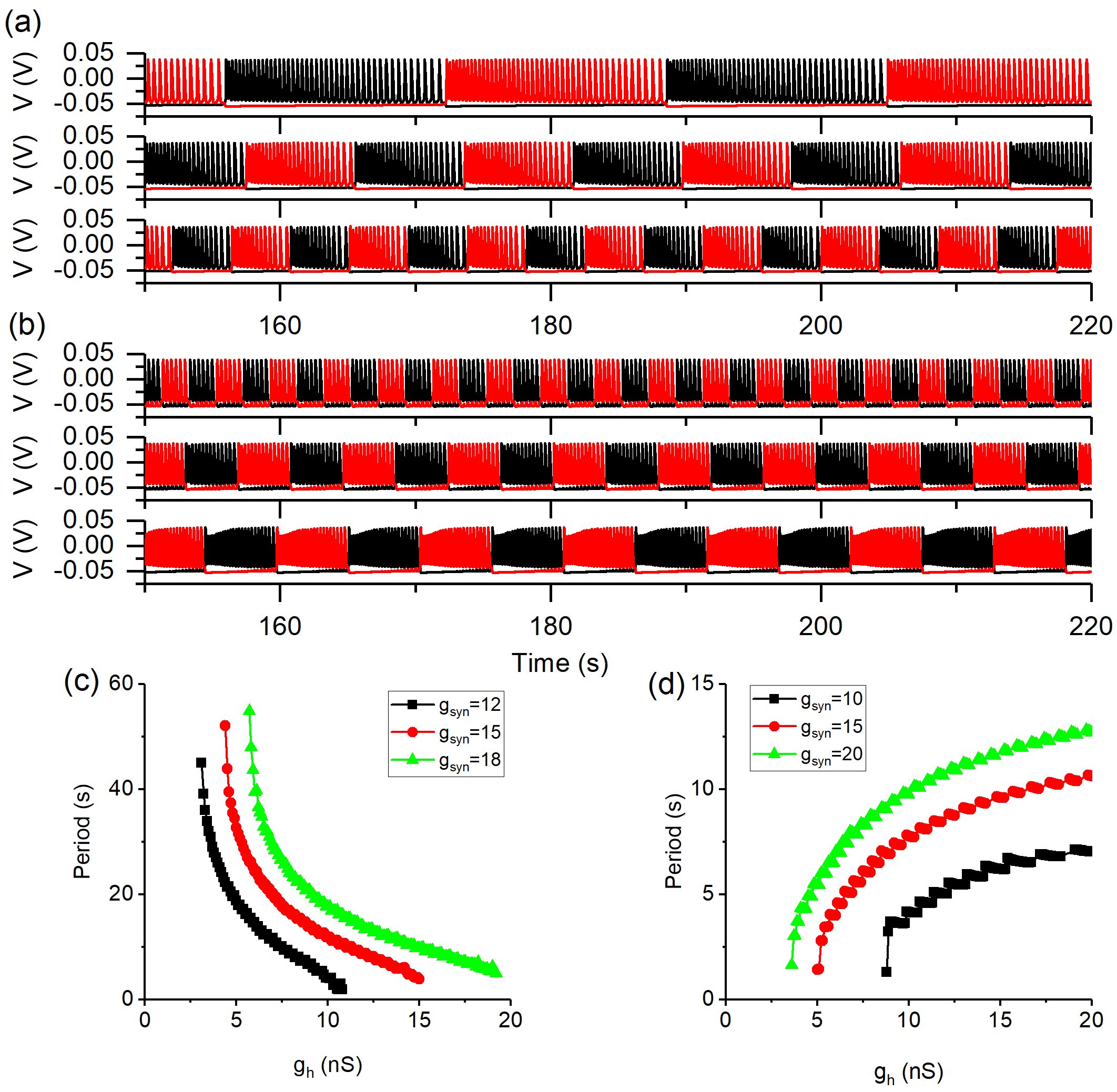

Supplement: Supplementary file 3 [file Data_Sheet_3.ZIP › S2.jpg]

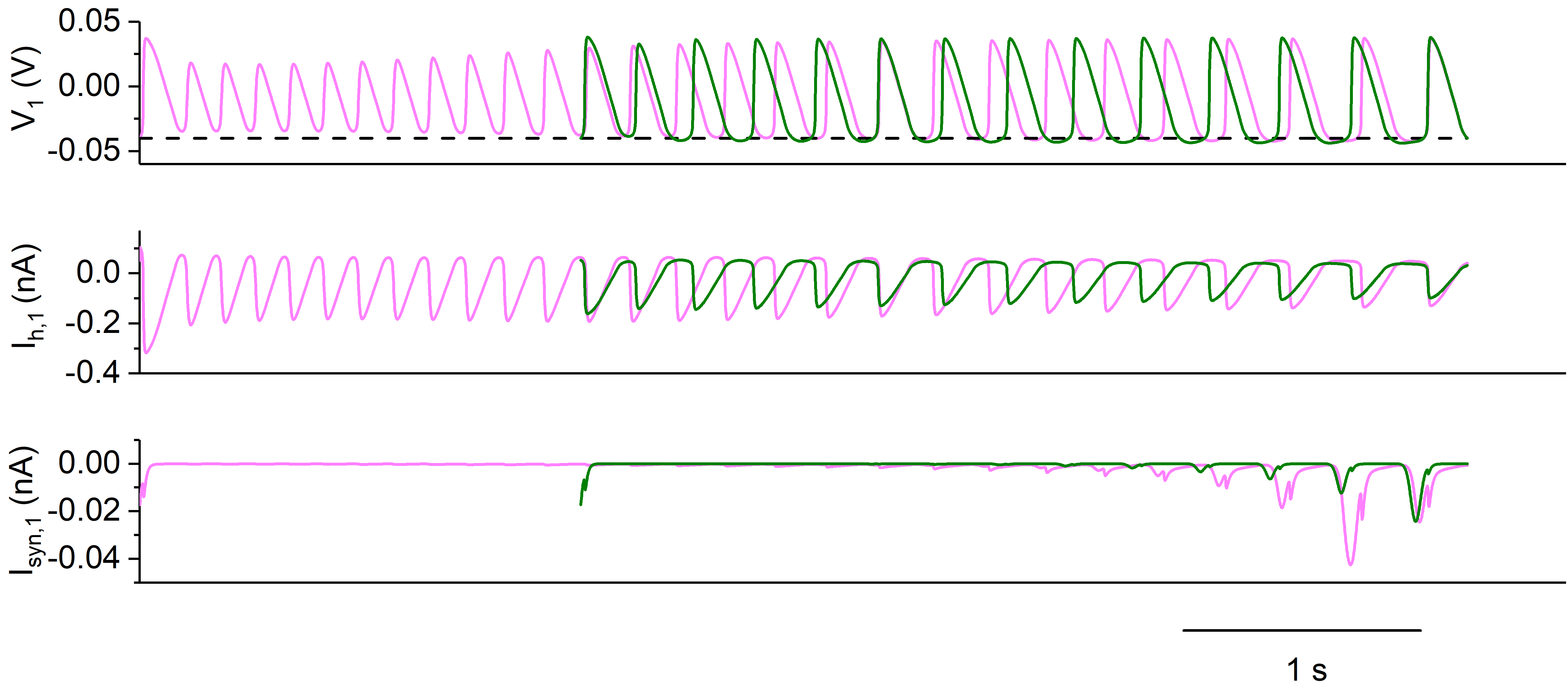

Supplement: Supplementary file 3 [file Data_Sheet_3.ZIP › S3.jpg]

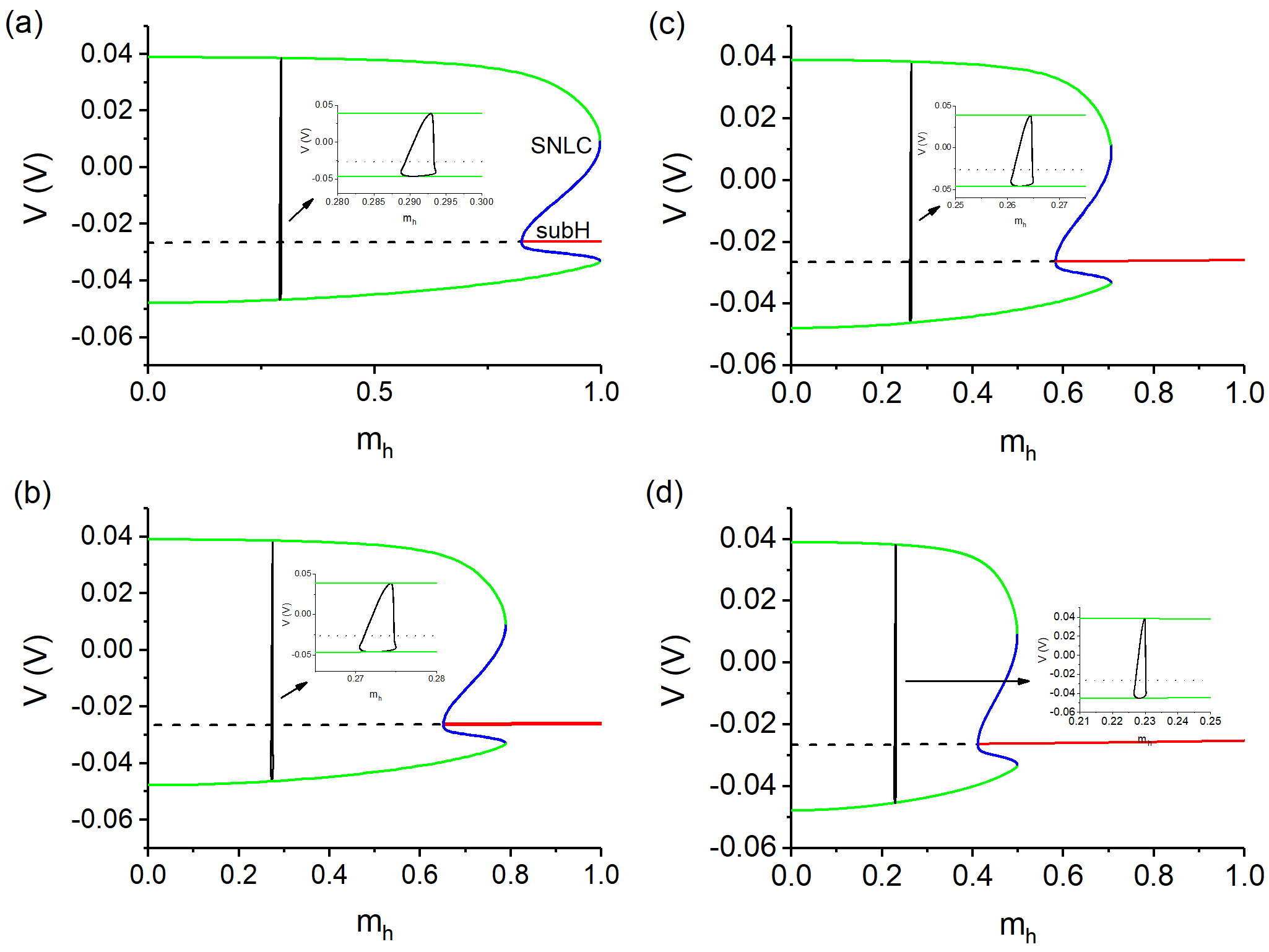

Supplement: Supplementary file 3 [file Data_Sheet_3.ZIP › S4.jpg]

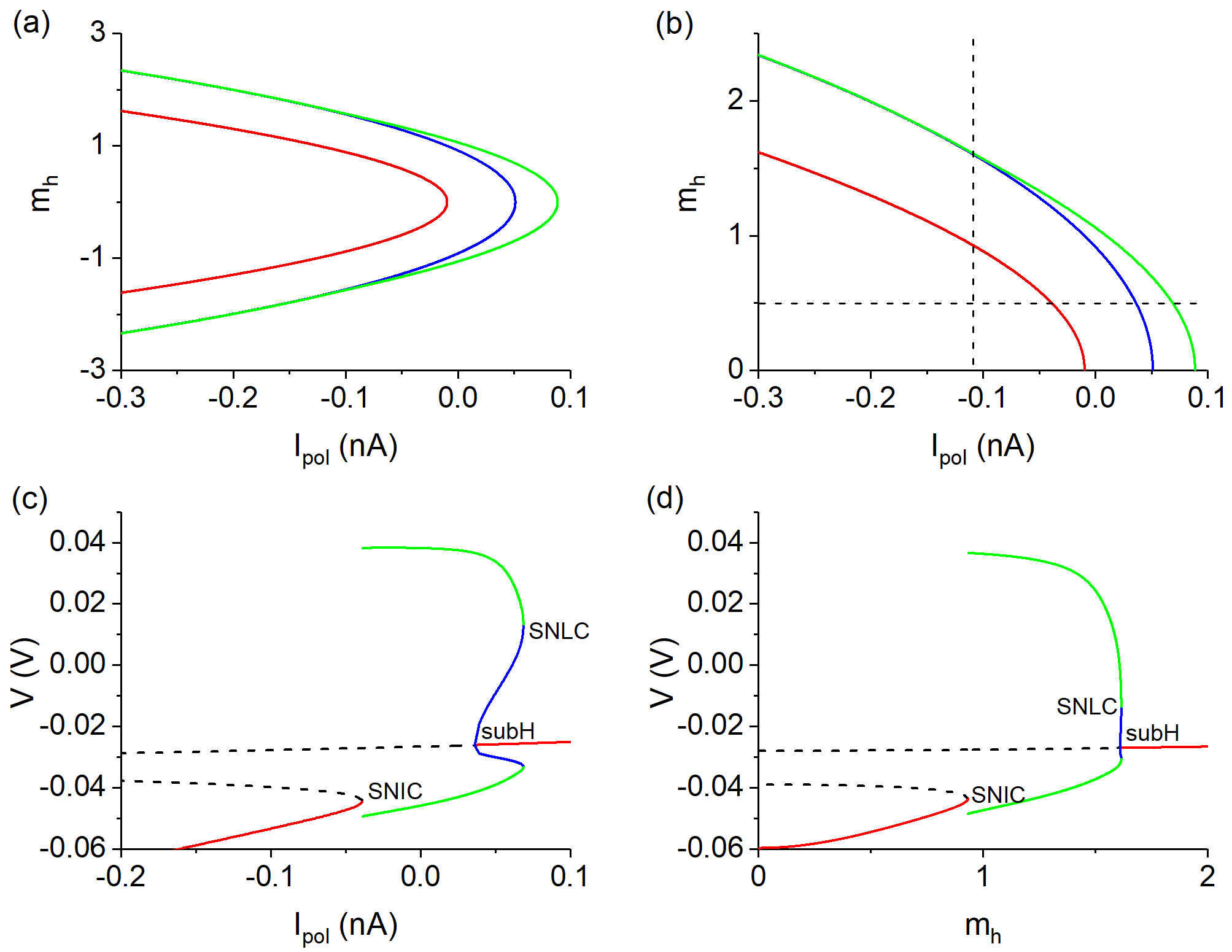

Supplement: Supplementary file 3 [file Data_Sheet_3.ZIP › S5.jpg]

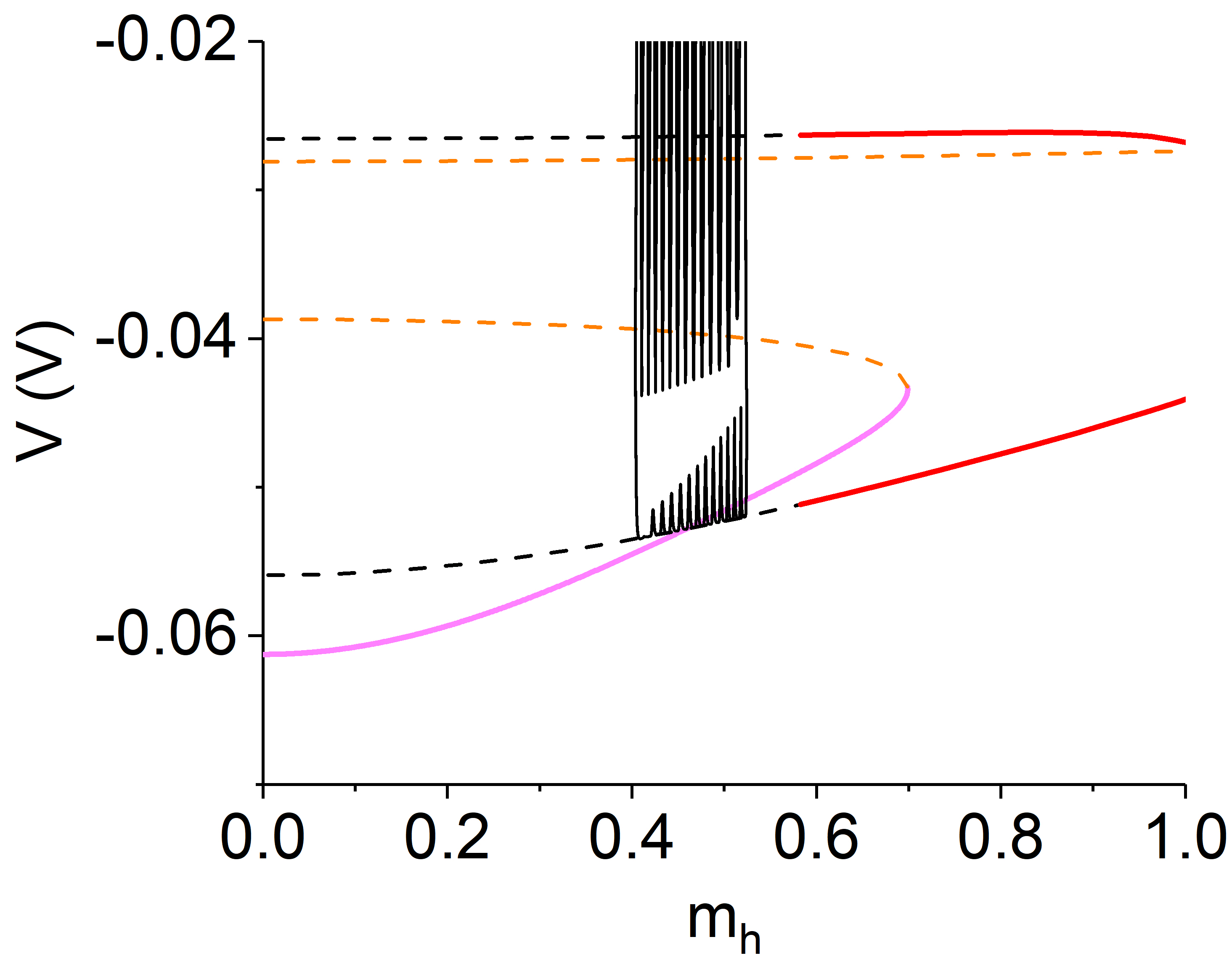

Supplement: Supplementary file 3 [file Data_Sheet_3.ZIP › S6.jpg]
